# Parameter Estimation for Kinetic Models of Chemical Reaction Networks from Partial Experimental Data of Species’ Concentrations

**DOI:** 10.3390/bioengineering10091056

**Published:** 2023-09-07

**Authors:** Manvel Gasparyan, Shodhan Rao

**Affiliations:** 1 School of Environmental Engineering, University of Seoul, Seoul 02504, Republic of Korea; 2 Center for Biosystems and Biotech Data Science, Ghent University Global Campus, Incheon 21985, Republic of Korea; shodhan.rao@ghent.ac.kr; 3 Department of Data Analysis and Mathematical Modelling, Ghent University, 9000 Ghent, Belgium

**Keywords:** systems biology, mathematical modeling, mass action kinetics, model reduction, least squares optimization, parameter identifiability

## Abstract

The current manuscript addresses the problem of parameter estimation for kinetic models of chemical reaction networks from observed time series partial experimental data of species concentrations. It is demonstrated how the Kron reduction method of kinetic models, in conjunction with the (weighted) least squares optimization technique, can be used as a tool to solve the above-mentioned ill-posed parameter estimation problem. First, a new trajectory-independent measure is introduced to quantify the dynamical difference between the original mathematical model and the corresponding Kron-reduced model. This measure is then crucially used to estimate the parameters contained in the kinetic model so that the corresponding values of the species’ concentrations predicted by the model fit the available experimental data. The new parameter estimation method is tested on two real-life examples of chemical reaction networks: nicotinic acetylcholine receptors and Trypanosoma brucei trypanothione synthetase. Both weighted and unweighted least squares techniques, combined with Kron reduction, are used to find the best-fitting parameter values. The method of leave-one-out cross-validation is utilized to determine the preferred technique. For nicotinic receptors, the training errors due to the application of unweighted and weighted least squares are 3.22 and 3.61 respectively, while for Trypanosoma synthetase, the application of unweighted and weighted least squares result in training errors of 0.82 and 0.70 respectively. Furthermore, the problem of identifiability of dynamical systems, i.e., the possibility of uniquely determining the parameters from certain types of output, has also been addressed.

## 1. Introduction

An important step in understanding the dynamics of a chemical reaction network (CRN) is its mathematical modelling. The dynamics of a CRN are usually determined by a system of ordinary differential equations (ODEs) known as the *kinetic model* of the CRN. The set of parameters of such a system is usually partially or even entirely unknown and is often estimated from various types of observational data obtained from biochemical experiments. Typically, the experimental data available for estimating the parameters are time series, i.e., the data are collected at discrete points in time.

The *bottom-up approach* (see, e.g., [1,2]) is a modelling method widely utilized in various research domains, including systems biology. In this method, accessible experimental data are employed to construct a comprehensive mathematical model of a system. The bottom-up modelling technique in systems biology comprises four primary stages. The initial stage is draft reconstruction, which involves the compilation of appropriate data from biological experiments. The second stage involves manually curating the gathered data, for example, by inserting absent values and removing irrelevant data. In the third stage, the knowledge concerning the biological interactions occurring in the CRN is transcribed into a mathematical expression. In the fourth stage, the parameters of this mathematical expression are numerically approximated from the observed experimental data, culminating in a comprehensive mathematical model.

The final stage of the bottom-up approach, namely parameter determination, can be executed using various approaches. The fundamental concept behind any method for parameter estimation is to compare the available experimental data with the corresponding values produced by the mathematical model. To guarantee a well-defined parameter estimation problem, it is essential to possess complete experimental data of external variables. This means having data corresponding to measurements of all external variables in the mathematical model. In the instance of CRNs, the choice of the most suitable technique generally relies on the qualities of the data amassed from biological experiments and the structure of the CRN being considered. Several mathematical techniques have been explored in the literature for parameter estimation of kinetic models of CRNs utilizing experimental data of species concentrations. A prevalent approach to solve the parameter estimation problem for scenarios where all species concentrations can be experimentally measured is the well-known (weighted) *least squares technique* (see, e.g., [3,4]). The application of this optimization approach minimises the (weighted) summation of squared *residuals*, i.e., the (weighted) summation of squared differences between the observed experimental concentration values and the corresponding values foreseen by the model. Some of the widely recognized methodologies such as maximum likelihood estimation, finite differences, quasi-linearization procedure, etc. have been deliberated in [5]. For an exhaustive overview of available mathematical techniques, consult [6,7,8,9]. In specific instances, experiments provide data corresponding to reaction rates (see, e.g., [10,11,12,13]). Recently, in [14], a method for parameter estimation from this form of experimental data for enzymatic CRNs was proposed. It is based on the approximation of the vector of species concentrations with parametric Bézier curves [15,16], which, in combination with the general least squares approach, leads to a complete mathematical model.

Bayesian-based parameter estimation techniques have also been widely developed for systems of ODEs (see, e.g., [17,18,19,20,21,22]). In such cases, the vector of parameters involved in the system of ODEs is usually treated as a random variable. A probability distribution (known as *prior distribution*) such as the normal distribution, the uniform distribution, the Poisson distribution, etc., (see, e.g., [23]) is therefore assigned to the vector of parameters. The technical core of the Bayesian parameter estimation approach is to construct the *joint probability distribution* (for the vector of parameters and the available data corresponding to the vector of dependent variables) and to perform computations to determine the *posterior distribution* (the conditional distribution of the parameters, given the experimental data corresponding to the dependent variable). Even though Bayesian-based approaches are useful techniques for parameter estimation and are extensively applied to CRNs (see, e.g., [24,25,26,27,28]), such approaches have many shortcomings. One of the main shortcomings is that most of these methods use only the available experimental data and do not consider, for the most part, the structure of the system of ODEs. Moreover, such an approach is mostly not straightforward to apply and may require huge computational efforts. Another shortcoming is the ambiguity in the construction of appropriate probability specifications since there is no explicit strategy for properly assigning a prior distribution. Depending on the characteristics of the available experimental data, it cannot always be assured that the model-predicted values corresponding to the obtained Bayesian estimates will be a satisfactory fit for the available experimental data. In such cases, a separate analysis of the fit of the model is required.

In most cases, not all concentrations can be measured experimentally. This incompleteness of the data makes the problem of parameter estimation more challenging, both mathematically and computationally, as we do not have a well-posed parameter estimation problem in this case. As expected, there is no general direct solution to this problem in the literature. Therefore, new mathematical techniques are necessary for estimating the parameters included in mathematical models using this type of data. In this manuscript, we address the problem of parameter estimation for CRNs from observed time series partial experimental data of species’ concentrations. We are mainly interested in CRNs governed by the *mass action kinetic rate law* (MAKRL), since this is the law that governs most real-life CRNs. We assume that measurements of concentrations are available for some of the species at discrete time points, which may not necessarily be equidistant. Direct estimation of the parameters of the mathematical model using the available partial experimental data is usually not feasible. This is because we do not have a well-posed parameter estimation problem, since not all concentrations involved in the mathematical model are measured experimentally. Therefore, we employ the *Kron reduction method* [29] to transform the ill-posed parameter estimation problem into a well-posed problem.

Before performing parameter estimation, it is important to understand whether the parameters are identifiable given a particular type of output, i.e., whether there is a unique parameter vector corresponding to the given output vector. We address the parameter identifiability issue of dynamical systems described by a system of ODEs. The uniqueness of the parameter vector depends on the structure of the system under consideration and the type of the available outputs. First, we recall two known definitions of parameter identifiability and then demonstrate the link between them

Our parameter estimation technique consists of three major steps. The first of these involves reduction of the known original model of the biochemical reaction network with unknown parameters using the technique of Kron reduction [29]. Of the several available techniques for model reduction of CRNs in the literature (see [30,31] for a thorough review of such techniques), we chose Kron reduction method for two particular reasons. The first and foremost is that Kron reduction preserves the kinetics of the original model. Thus, if the original network is governed by MAKRL, the reduced model obtained by Kron reduction also corresponds to another CRN, whose variables are concentrations of chemical species that are a subset of the species of the original network and moreover, the reduced CRN is also governed by MAKRL. This kinetics preservation property does not hold for several other known reduction techniques. The second reason is that by using Kron reduction method, it is possible to arrive at a reduced model of a CRN, whose dependent variables are exactly the set of concentrations of compounds whose time-series data are available. The (unknown) parameters of this reduced model are functions of the parameters of the original mathematical model. The availability of this reduced model with unknown parameters together with the time-series data of all the dependent variables of this model leads to a well-posed parameter estimation problem. The solution of this parameter estimation problem is the second step of our procedure. Because of its simplicity and computational feasibility, we apply the least squares optimization technique to deal with this estimation problem. In the final step, we solve an optimization problem in which the parameters of the original model are determined in such a way that the original mathematical model and the Kron reduced model have minimum difference between a key characteristic property associated with them. It is shown that this key characteristic property is related to the settling time of the corresponding CRN in case the given model is linear. The entire procedure has been automated and the corresponding MATLAB library is provided as Supplementary Material.

We apply our new techniques to two realistic examples of CRNs from the *Biomodels database* [32]. We consider a model of *nicotinic acetylcholine receptors* [33] and a model of *Trypanosoma brucei trypanothione synthetase* [34]. For each of these models, we first generate partial time series data of the species’ concentrations using the parameter values given in the corresponding reference. We then explain how to derive the Kron-reduced mathematical model obtained by deleting a subset of complexes and determine the values for its parameters such that the dynamics of the Kron-reduced model in the least squares sense most closely approximate the available time series data. Using these estimated values for the parameters of the Kron-reduced model and their dependence on the parameters of the original mathematical model, we finally determine the estimates for the parameters contained in the original model.

## 2. Background

In this section, we give a compact description of the mathematical tools that are necessary for demonstrating the main results of the current manuscript.

### 2.1. Notations

We introduce the notations that will be used throughout the rest of the manuscript. For a vector $v\in R^{m}$, $v_{i}$ refers to its *i*th entry, i.e., $v={\left[v_{i}\right]}_{i=1}^{m}$. $\Gamma_{v}=\mathrm{diag}\left(v\right)$ denotes the m×m diagonal matrix, whose diagonal entries are the entries of the vector *v*. $M_{ij}$ is the entry of the matrix *M* corresponding to the *i*th row and the *j*th column. The eigenvalues of the m×m square matrix *M* are denoted by $\lambda_{1}\left(M\right),\ldots ,\lambda_{m}\left(M\right)$. The spectrum of the square matrix *M* is denoted by σ(M), i.e., $\sigma \left(M\right)=\left\{\lambda_{i}\left(M\right);i=1,\ldots ,m\right\}$. $\bar{u}$ is the complex conjugate of the complex number u∈C. ℜ(u) and ℑ(u) denote the real and imaginary parts of the complex number C, respectively. |I| is the cardinality of the set I. Vectors of length *m* composed entirely of ones and zeros are respectively denoted as ${𝟙}_{m}$ and ${𝟘}_{m}$. Furthermore, ${𝟘}_{m\times n}$ is the m×n matrix with all its entries set to zero.

### 2.2. Mathematical Models

We outline the process of deriving a mathematical formulation that describes the dynamics of a CRN. Let $X_{i}$, i=1,…,s, be the set of *s* distinct chemical species of the considered CRN with *r* unidirectional reactions $R_{j}$, j=1,…,r. The connection between the species and the reactions is established through an s×r stoichiometric matrix denoted as *S*. Its elements are determined as $S_{ij}=\beta_{ji}-\alpha_{ji}$, with $\alpha_{ji}$ representing the number of moles of the *i*th species $X_{i}$ within the substrate of the *j*th reaction $R_{j}$, and $\beta_{ji}$ indicating the same for the product of the reaction. Denote by $\nu \in R_{+}^{r}$ the vector of unidirectional reaction rates. This vector is dependent on both the species’ concentration vector *x* and the parameter vector $\kappa \in R_{+}^{q}$ inherent to the model. The fundamental framework that characterizes the evolution of the species concentration vector is given by the stoichiometric representation of the *balance laws* as:

(1) $$\frac{dx}{dt}=S\nu \left(\kappa ,x\right).$$

Graphs are crucial tools for modelling various types of relations and processes in numerous scientific domains including systems biology. In chemical reaction network theory (CRNT), directed graphs are commonly used in the process of modelling CRNs to display the link between individual reactions. The *complexes* of a CRN are defined as the left-hand (substrate) and right-hand (product) sides of the reactions. Let $C_{n}$, n=1,…,c, denote the set of distinct complexes of the considered CRN. The complexes can be inherently linked with the vertices of a directed graph, where the directed edges align with the reactions present within the CRN. More precisely, if there is a reaction for which the complex $C_{i}$ is the substrate and the complex $C_{j}$ is the product, then in the corresponding graph of complexes there is a directed edge having the vertex associated with the complex $C_{i}$ as the tail vertex and the vertex associated with the complex $C_{j}$ as the head vertex. A *linkage class* of a CRN is a connected component of the corresponding graph of complex, i.e., a maximal set of complexes such that every complex in the set is connected by a directed edge to at least one other complex.

Any CRN can be uniquely described by a system of ODEs given in (1), independent of its governing laws. In this manuscript, we consider only mass action kinetics rate law, since it is the governing law of a wide range of real-life CRNs. According to this rate law, the rate of a reaction is directly proportional to the concentration of each species involved in the substrate of the reaction, raised to a power equal to the number of its moles in the expression of this substrate. More precisely, the reaction rate $\nu_{j}$ of the *j*th reaction, j=1,…,r, is given in the following form

(2) $$\nu_{j}=k_{j}\prod_{i=1}^{s}x_{i}^{\alpha_{ij}},$$

where, as earlier, $k_{j}$, j=1,…,r, is the rate constant of the *j*th reaction and $\alpha_{ij}$, i=1,…,s, is the number of moles of the species $X_{i}$ in the substrate of the *j*th reaction. Note that in this case, the only parameters contained in the mathematical model are the rate constants of the reactions, i.e., κ=k and the number of unknown parameters is q=r. Next, we obtain an expression for the vector of reaction rates $\nu \in R_{+}^{r}$ given in terms of matrix multiplication, which is a useful approach for automated modelling purposes. Define the s×r the *substrate composition matrix*Ω and the *substrate expression function*$\varphi :R_{+}^{s}\to R_{+}^{r}$ of the CRN as:

Ωij=−Sij,ifSij<0−S0,otherwise,φ(x)=∏i=1sxiΩijj=1r.

The *conductance matrix* $\Gamma_{k}$ of the CRN is a r×r diagonal matrix whose *i*th diagonal entry is the rate constant of the *i*th reaction $R_{i}$, i.e., if $k\in R_{+}^{r}$ is the vector of rate constants, then $\Gamma_{k}=\mathrm{diag}\left(k\right)$. Then observe that the vector of reaction rates can be expressed in the following matrix multiplication form:

(3) $$\nu \left(k,x\right)=\Gamma_{k}\varphi \left(x\right).$$

**Example** **1.** *To elucidate the outlined modelling process, we apply it to the subsequent example of a CRN. Consider a scenario where five chemical species, denoted as $X_{i}$, for i=1,…,5, are engaged in three distinct unidirectional reactions, given as follows:*

(4) $$X_{1}+X_{2}\;\;⇌\;\;X_{3}\;\;⟶\;\;X_{4}+X_{5}.$$

*For i=1,…,3, let $k_{i}$ be the rate constant of the ith reaction. Observe that the second reaction can be interpreted as the reverse of the first reaction. In our modelling approach, we treat each reversible reaction as a pair of distinct unidirectional reactions. There are three distinct complexes $C_{i}$, i=1,…,3, involved in the CRN, which are given as $C_{1}=X_{1}+X_{2}$, $C_{2}=X_{3}$, and $C_{3}=X_{4}+X_{5}$. For the first reaction, since the complex $C_{1}$ is the substrate and the complex $C_{2}$ is the product, in the graph of complexes corresponding to the CRN shown in (4) there is a directed edge having $C_{1}$ as the tail vertex and $C_{2}$ as the head vertex. Similarly, we can construct the edges of the graph of complex corresponding to the other reactions. The resulting graph of complexes is thus $C_{1}⇌C_{2}\to C_{3}$. Note that this graph of complexes consists of only a single linkage class.*

*Given the assumption that the reactions (4) are governed by MAKRL, the reaction rates can be computed by Equation (2) as follows:*

$$\nu_{1}=k_{1}x_{1}x_{2},\;\nu_{2}=k_{2}x_{3},\;\nu_{3}=k_{3}x_{3}.$$

*The vector of reaction rates can be written in the matrix multiplication form (3) with the substrate composition matrix* Ω *and the substrate expression function φ given by*

$$\begin{array}{ccc} \Omega =\left[\begin{array}{ccc} 1 & 0 & 0\\ 1 & 0 & 0\\ 0 & 1 & 1\\ 0 & 0 & 0\\ 0 & 0 & 0 \end{array}\right], & & \varphi \left(x\right)=\left[\begin{array}{c} x_{1}x_{2}\\ x_{3}\\ x_{3} \end{array}\right]. \end{array}$$

*Thus, in this case the balance laws (1) can be written as:*

(5) $$\begin{array}{c} \begin{array}{cc} & \frac{dx_{1}}{dt}=\frac{dx_{2}}{dt}=-k_{1}x_{1}x_{2}+k_{2}x_{3},\\ & \frac{dx_{3}}{dt}=k_{1}x_{1}x_{2}-k_{2}x_{3}-k_{3}x_{3},\\ & \frac{dx_{4}}{dt}=\frac{dx_{5}}{dt}=k_{3}x_{3}. \end{array} \end{array}$$

### 2.3. The Weighted Directed Laplacian Matrix

In CRNT, the (*weighted directed*) *Laplacian matrix* is a matrix representation of the reactions occurring between the different complexes. Here we explain how to construct the Laplacian matrix of the CRN using the (*weighted directed*) *adjacency matrix* corresponding to its graph of complexes. The adjacency matrix *A* is a c×c matrix, with *c* being the number of complexes of the CRN, such that its entry $A_{ij}$ is equal to *k* if there is a reaction having the *j*th complex of the network as substrate and *i*th complex of the network as a product with *k* being the rate constant of the reaction. The (*weighted directed*) *degree matrix D* of the graph of complexes corresponding to a CRN is a c×c diagonal matrix such that its *i*th diagonal entry is equal to the sum of the elements of the *i*th column of the weighted adjacency matrix *A*. The c×c Laplacian matrix associated with the graph of complexes of the considered CRN is defined as follows:

L=D−A.

If there is a reaction for which the complex $C_{j}$ is the substrate and the complex $C_{i}$ is the product, then the off-diagonal element $L_{ij}$ is equal to the rate constant of the respective reaction taken with the negative sign. For useful properties of Laplacian matrices, we refer to [35]. Any directed graph is defined by an *incidence matrix* [36], which represents the connections between its vertices and edges. In the case of CRNs, the c×r incidence matrix *B* of the graph of complexes is defined as follows:

(6) Bij=−1,ifthecomplexCiisthesubstrateofthereactionRj,−1,ifthecomplexCiistheproductofthereactionRj−0,otherwise.

Define the c×r *outgoing matrix* Δ of the considered CRN as follows:

$$\Delta_{ij}=\left\{\begin{array}{cc} 0, & \mathrm{if}\;B_{ij}=1\\ B_{ij}, & \mathrm{otherwise}. \end{array}\right.$$

It can be shown that the Laplacian is given in matrix multiplication form as follows:

(7) $$L=B\Gamma_{k}\Delta^{⊤}.$$

For automatic modelling purposes, it is convenient to construct the Laplacian matrix using the simple matrix multiplication form given in (7).

Next, we show how to represent the balance laws (1) in terms of the weighted directed Laplacian matrix *L*. The *c* complexes of the considered CRN are described by an s×c *complex composition matrix Z*, whose columns express the complexes of the CRN in terms of their species. More precisely, the element $Z_{ij}$ of the complex composition matrix *Z* is the number of moles of the *i*th species $X_{i}$ in the expression of *j*th complex $C_{j}$. As explained in [29,37], it can be shown that the balance laws of a mass action CRN can be rewritten as follows:

(8) $$\frac{dx}{dt}=-ZL\psi \left(x\right),$$

where $\psi :R_{+}^{s}\to R_{+}^{c}$ is the *complex expression function* defined as:

$$\psi \left(x\right)={\left[\prod_{i=1}^{s}{x_{i}}^{Z_{ij}}\right]}_{j=1}^{c}.$$

**Example** **2.** *With reference to the CRN example (4), the 3×3 weighted adjacency matrix A and the 3×3 weighted degree matrix D are:*

$$\begin{array}{c} A=\left[\begin{array}{ccc} 0 & k_{2} & 0\\ k_{1} & 0 & 0\\ 0 & k_{3} & 0 \end{array}\right],\;D=\left[\begin{array}{ccc} k_{1} & 0 & 0\\ 0 & k_{2}+k_{3} & 0\\ 0 & 0 & 0 \end{array}\right]. \end{array}$$

*The 3×3 weighted directed Laplacian L of the CRN (4) is therefore given by*

L=−k1−k20−k1k2+k30−0−k30,

*On the other hand, the Laplacian matrix can be computed using Equation (7) with the incidence matrix B and the outgoing matrix* Δ *given by:*

B=−1−1−0−1−1−1−0−0−1,Δ=−1−0−0−0−1−1−0−0−0.

*The balance laws (5) can be rewritten as Equation (8) with the 5×3 complex composition matrix Z and the complex expression function ψ given by:*

$$\begin{array}{c} Z=\left[\begin{array}{ccc} 1 & 0 & 0\\ 1 & 0 & 0\\ 0 & 1 & 0\\ 0 & 0 & 1\\ 0 & 0 & 1 \end{array}\right],\;\psi \left(x\right)=\left[\begin{array}{c} x_{1}x_{2}\\ x_{3}\\ x_{4}x_{5} \end{array}\right]. \end{array}$$

In the following theorem we recall certain important spectral properties (see, e.g., [38]) of the weighted directed Laplacian matrix associated with the graph of complexes corresponding to a CRN governed by MAKRL.

**Theorem** **1** (Spectrum of the weighted directed Laplacian matrix). *If the graph of complexes of a CRN governed by MAKRL has a single linkage class, then the eigenvalues $\lambda_{i}$, i=1,…,c, of the weighted directed Laplacian matrix L associated with the CRN can be ordered in such a way that:*

(9) $$\begin{array}{c} 0=\lambda_{1}<ℜ\left(\lambda_{2}\right)\leq ℜ\left(\lambda_{3}\right)\leq \ldots \leq ℜ\left(\lambda_{c}\right). \end{array}$$

First note that 0∈σ(L). This is simply because of the fact that the sum of each column of *L* is equal to zero according to the definition of the Laplacian matrix, i.e., $L^{⊤}{𝟙}_{c}={𝟘}_{c}$. Moreover, from the generalization of the matrix-tree theorem it follows that the multiplicity of the zero eigenvalue is equal to the number of connected components, which is c−rank(L). Using Greshgorin’s circle theorem [39], it can be shown that the real parts of non-zero complex eigenvalues of *L* are strictly positive, i.e., if λ∈σ(L) and λ≠0, then ℜ(λ)>0. For a detailed explanation of the proof of Theorem 1 we refer to [40].

### 2.4. Kron Reduction of Chemical Reaction Networks

The Kron reduction for mathematical models of CRNs [29,37,41] is performed by assuming that certain intermediate complexes are complex balanced and is carried out by computing the Schur complement of the weighted Laplacian matrix associated with the corresponding graph of complexes. We therefore first recall the definition of Schur complements (see, e.g., [42]) of a given square matrix.

**Definition** **1** (Schur complement). *Let $A_{1}\in R^{n\times n}$, $A_{2}\in R^{n\times m}$, $A_{3}\in R^{m\times n}$, and $A_{4}\in R^{m\times m}$ be constant matrices such that the latter is invertible. Consider the following (n+m)×(n+m) block matrix:*

$$L=\left[\begin{array}{cc} A_{1} & A_{2}\\ A_{3} & A_{4} \end{array}\right].$$

*The Schur complement of the block matrix $A_{4}$ is the n×n matrix $\hat{L}$ defined as:*

$$\hat{L}=A_{1}-A_{2}A_{4}^{-1}A_{3}.$$

Let I be the set of indices corresponding to the complexes of the CRN, i.e., I={1,…,c}. Suppose our objective involves removing the complexes associated with the subset of indices denoted as $\bar{I}$. Note that it should be ensured that $|\bar{I}|<c$. The removal of complexes is accomplished through the computation of the Schur complement $\hat{L}\in R^{|\hat{I}\left|\times \right|\hat{I}|}$ of the block matrix of the Laplacian matrix *L* corresponding to the set of indices $\bar{I}$. Here, $\hat{I}=I\setminus \bar{I}$ is the set of indices corresponding to the complexes remaining in the reduced graph of complexes. $\hat{L}$ is again a Laplacian matrix since it satisfies the properties of Laplacian matrices ([29], Proposition 1). Furthermore, it has been proven that the equation

$$\frac{dy}{dt}=-\hat{Z}\hat{L}\hat{\psi }\left(y\right),$$

describes the dynamics of a CRN governed by MAKRL, with a smaller number of complexes. Here, $y\in R_{+}^{s}$ is the vector of species’ concentrations in the reduced mathematical model (which contains a subset of the elements of *x*.), $\hat{Z}$ is the complex composition matrix of the reduced CRN, and

$$\hat{\psi }\left(y\right)={\left[\prod_{i=1}^{s}y_{i}^{{\hat{Z}}_{ij}}\right]}_{j=1}^{|\hat{I}|}.$$

As explained in [29,37], a well-chosen $\bar{I}$ will result in a reduction of dependent variables within the corresponding mathematical model. Note that the parameters contained in the Kron-reduced mathematical model can be represented as a function of the parameters involved in the original model. More precisely, if $p\in R_{+}^{\hat{r}}$ denotes the vector of parameters of the reduced model with $\hat{r}$ being the number of reactions in it, then there is a function $f:R_{+}^{r}\to R_{+}^{\hat{r}}$ such that p=f(k). In general, the manual derivation of the explicit form of the function *f* is not straightforward. However, we use MATLAB symbolic variables to derive the explicit form of *f* in a fully automated fashion. We refer to the function *f* as the *parameter dependence function*, since it specifies the dependence of the vector of parameters *p* of the reduced model on the vector of parameters *k* of the original model.

In order to determine the structure of the reduced CRN, we need to find the incidence matrix and the complex composition matrix of the reduced network. This can be done according to the automated procedure described in [37]. The incidence matrix $\hat{B}$ is determined by making use of its Laplacian matrix $\hat{L}$. According to this procedure, if ${\hat{L}}_{ij}<0$, i≠j, then in the reduced graph of complexes there is a reaction for which the *j*th complex of the reduced CRN is the substrate and *i*th complex of the reduced CRN is the product complex. Therefore, the entries of the incidence matrix $\hat{B}$ of the reduced graph of complexes are defined according to (6). The complex composition matrix $\hat{Z}$ of the reduced CRN is obtained by simply removing the columns of the incidence matrix *Z* of the original CRN that correspond to the set of indices $\bar{I}$. As mentioned earlier, the incidence matrix describes the reactions occurring between the complexes and the complex composition matrix gives the expression of complexes in terms of the species. We therefore use $\hat{B}$ and $\hat{Z}$ to determine the reactions corresponding to the reduced graph of complexes.

**Example** **3.** *To illustrate the Kron reduction method for CRNs, we demonstrate it for the example given in (4). Assume that we want to delete the complex $C_{2}$ from the graph of complexes by applying the Kron reduction method. In other words, $\bar{I}=\left\{2\right\}$ and $\hat{I}=\left\{1,3\right\}$. The elimination of $C_{2}$ is carried out by computing the Schur complement of the Laplacian matrix corresponding to the set of indices $\bar{I}$, which results in the following 2×2 weighted directed Laplacian matrix associated with the reduced graph of complexes:*

L^=−k1k3k2+k30−k1k3k2+k30.

*As explained in [37], the complex composition matrix $\hat{Z}$ of the reduced CRN is obtained by eliminating the second column of the complex composition matrix Z of the original CRN, i.e.,*

$$\hat{Z}=\left[\begin{array}{cc} 1 & 0\\ 1 & 0\\ 0 & 0\\ 0 & 1\\ 0 & 1 \end{array}\right]$$

*Thus the balance laws of the Kron-reduced model are as follows:*

dy1dt=dy2dt=−k1k3k2+k3y1y2,dy3dt=0,dy4dt=dy5dt=−k1k3k2+k3y1y2.

*Using the Laplacian matrix $\hat{L}$ of the Kron-reduced model we obtain the incidence matrix $\hat{B}$ of the reduced complex graph:*

B^=−1−1.

*Taking into account $\hat{Z}$ and $\hat{B}$ we derive the reactions of the reduced CRN:*

$$X_{1}+X_{2}\;\;\overset{p}{\to }\;\;X_{4}+X_{5},$$

*where the parameter p is given in terms of the parameters of the original model as $p=\frac{k_{1}k_{3}}{k_{2}+k_{3}}$, i.e., in this case for the explicit form of the function f we have $f\left(k\right)=\frac{k_{1}k_{3}}{k_{2}+k_{3}}$. Note that after deleting the complex $C_{2}$ from the graph of complexes by the Kron reduction approach, the species $X_{3}$ is not involved in the resulting reduced CRN, since its concentration $x_{3}$ is conserved in time.*

In [29,37], the optimal combination of complexes for deletion is selected by making use of an error integral, which quantifies the difference between the dynamical behaviors of the original model and the corresponding reduced model. This error integral is measure that is based on a particular trajectory corresponding to the original model.

### 2.5. The Least Squares Optimization Method

We explain how to apply the (weighted) least squares optimization technique to estimate the parameters $\kappa \in R_{+}^{p}$ involved in the kinetic model (1) describing the dynamics of the given CRN. For the general least squares optimization method we refer to, for example, [3,4]. This optimization method plays a crucial role in our parameter estimation method.

Assume that biological experiments provide complete experimental data of species’ concentrations, i.e., measurements that correspond to all of the species’ concentrations. For j=1,…,n, let ${\hat{x}}_{i,m}^{\left(j\right)}$, i=1,…,s; $m=0,\ldots ,N_{j}$, be the observed value of the *i*th concentration at time instant $t_{m}^{\left(j\right)}$, which is the *m*th time point corresponding to the *j*th experiment. We aim to identify the best-fitting parameter values of the mathematical model (1) corresponding to the above-mentioned observed time-series experimental data of species’ concentrations. For every j=1,…,n, consider the following initial value problem (IVP):

(10) $$\left\{\begin{array}{c} \frac{dx}{dt}=S\nu \left(\kappa ,x\right)\\ x\left(t_{0}^{\left(j\right)}\right)={\hat{x}}_{i,0}^{\left(j\right)} \end{array}\right..$$

Since the available experimental data corresponds to the measurements of all the species’ concentrations, for every parameter vector $\kappa \in R_{+}^{p}$ the IVP given in (10) generates data for the species’ concentrations. More precisely, the IVP (10) can be numerically solved with respect to time. However, this numerical integration is not always possible if the available experimental data corresponds to only some of the concentrations.

For every j=1,…,n, let $x_{i}\left(t_{m}^{\left(j\right)},\kappa \right)$, i=1,…,s; $m=0,\ldots ,N_{j}$, denote the model-predicted value of the *i*th concentration obtained by numerically solving the IVP (10). In this case, the least squares error is defined as the sum of squared residuals, which are the differences between the observed experimental values of concentrations and the corresponding model-predicted values provided from the IVP given in (10):

(11) $$\varepsilon \left(\kappa \right)=\sum_{j=1}^{n}\sum_{i=1}^{s}w_{i}^{\left(j\right)}\sum_{m=0}^{N_{j}}{\left(x_{i}\left(t_{m}^{\left(j\right)},\kappa \right)-{\hat{x}}_{i,m}^{\left(j\right)}\right)}^{2}.$$

Here, for i=1,…,s and j=1,…,n, $w_{i}^{\left(j\right)}$ denotes the weight of the corresponding measurement. In this case, it is assumed that the measurements have different uncertainties. Each weight can be taken, for example, equal to the reciprocal of the variance of the measurement:

$$\begin{array}{c} w_{i}^{\left(j\right)}=\frac{1}{{\left[\sigma_{i}^{\left(j\right)}\right]}^{2}},\;i=1,\ldots ,s;\;\;j=1,\ldots ,n. \end{array}$$

We will refer to this approach as the weighted least squares (WLS). If the measurements have equal variance, then the weights can be taken equal to one. We will refer to the corresponding approach as the unweighted least squares (UWLS). The (weighted) least squares optimization technique finds the optimal parameter values by minimizing the error (11). This minimization can be done, for example, by the standard Levenberg-Marquardt algorithm [43,44], or the modified Levenberg-Marquardt algorithm [45]. We denote by $\hat{\kappa }\in R_{+}^{p}$ the solution to the aforementioned optimization problem, i.e., $\hat{\kappa }=\arg\min_{\kappa }\varepsilon \left(\kappa \right).$

## 3. Parameter Identifiability

In this section, we first recall the definitions of least squares parameter identifiability and parameter identifiability, and in addition demonstrate the link between these two identifiability concepts. Consider a dynamical system described by a system of ODEs that is given in following the form:

(12) $$\begin{array}{c} \left\{\begin{array}{c} \frac{dx}{dt}=f\left(\kappa ,x\right)\\ y=g(\kappa ,x) \end{array}\right., \end{array}$$

where *f* is an *s*-dimensional vector-valued function depending on the structure of the system and *g* is *n*-dimensional. Here, $\kappa \in R_{+}^{p}$ is the parameter vector, $x:R_{+}\to R_{+}^{s}$ is the vector of states of the system and *y* is the *n*-dimensional output vector. For a given vector of initial states $x_{0}\in R_{+}^{s}$, let $y(t|\kappa ,x_{0})$ denote the output trajectory of the system (12) corresponding to the parameter vector $\kappa \in R_{+}^{p}$ and initial states $x_{0}\in R_{+}^{s}$.

Assume that, in an experimental setup, the output has been continuously measured over the time interval [0,T], for some pre-specified T>0. Let $\hat{y}:R_{+}\to R^{n}$ be the resulting measured output. Consider the cost function $\varepsilon_{x_{0}}:R_{+}^{p}\to R_{+}$ defined as:

(13) $$\begin{array}{c} \varepsilon_{x_{0}}\left(\kappa \right)=\sum_{i=1}^{n}\int_{0}^{T}{\left(y_{i}\left(t|\kappa ,x_{0}\right)-{\hat{y}}_{i}\left(t\right)\right)}^{2}dt, \end{array}$$

We recall the definition of least squares parameter identifiability of dynamical systems given in the form (12), which was first introduced in [46].

**Definition** **2** (Least squares parameter identifiability). *The dynamical system (12) is least squares parameter identifiable, if for every given vector of initial states $x_{0}$ and for every given measurement function $\hat{y}$, the cost function (13) admits a unique minimum.*

If there exists at least one vector of initial states $x_{0}$ and a measurement function $\hat{y}$ such that the cost function (13) has multiple minima, then the dynamical system (12) is least squares parameter nonidentifiable.

Each vector of parameters $\kappa \in R_{+}^{p}$ determines a set $Y_{\kappa }$ of admissible output trajectories of the system (12). We recall the definition of parameter identifiability of dynamical systems provided in [14].

**Definition** **3** (Parameter identifiability). *The dynamical system (12) is parameter identifiable, if for parameter vectors $\kappa ,\bar{\kappa }\in R_{+}^{p}$ such that $\kappa \neq \bar{\kappa }$, we have $Y_{\kappa }\neq Y_{\bar{\kappa }}$.*

Equivalently, the dynamical system (12) is parameter identifiable, if $Y_{\kappa }=Y_{\bar{\kappa }}$ for two parameter vectors $\kappa ,\bar{\kappa }\in R_{+}^{p}$, implies $\kappa =\bar{\kappa }$. If there are two distinct parameter vectors $\kappa \neq \bar{\kappa }$ for which $Y_{\kappa }=Y_{\bar{\kappa }}$, then the dynamical system (12) is parameter unidentifiable.

We finally turn our attention to the main contribution of this section. In the following theorem we specify a link between the two identifiability concepts given above.

**Theorem** **2.** *If the dynamical system (12) is parameter unidentifiable, then it is also least squares parameter unidentifiable.*

**Proof.**  We assume that the dynamical system (12) is parameter unidentifiable and we prove that it is also least squares parameter unidentifiable. Since the dynamical system (12) is parameter unidentifiable, there are two parameter vectors $\kappa ,\bar{\kappa }\in R_{+}^{p}$, such that $\kappa \neq \bar{\kappa }$ and $Y_{\kappa }=Y_{\bar{\kappa }}$. This means that there is at least one vector of initial states $x_{0}\in R_{+}^{s}$ for which $y\left(t|\kappa ,x_{0}\right)=y\left(t|\bar{\kappa },x_{0}\right)$, t∈[0,T]. Choose a measurement function $\hat{y}:R_{+}\to R^{n}$ as:

$$\begin{array}{c} \hat{y}\left(t\right):=y\left(t|\kappa ,x_{0}\right)=y\left(t|\bar{\kappa },x_{0}\right),\;t\in \left[0,T\right]. \end{array}$$

Note that this choice results in $\varepsilon_{x_{0}}\left(\kappa \right)=\varepsilon_{x_{0}}\left(\bar{\kappa }\right)=0$. For two parameter vectors $\kappa \neq \bar{\kappa }$ there is a vector of initial states $x_{0}\in R_{+}^{s}$ and a measurement function $\hat{y}:R_{+}\to R^{n}$ such that $\varepsilon_{x_{0}}\left(\kappa \right)=\varepsilon_{x_{0}}\left(\bar{\kappa }\right)=0$. Note that zero is the minimum value of $\varepsilon_{x_{0}}$ since it is a non-negative function. We conclude that the minimum of $\varepsilon_{x_{0}}$ is not unique in this case and thus the dynamical system (12) is least squares parameter unidentifiable. □

## 4. Parameter Estimation Procedure

In this section, we describe the main contribution of the current manuscript. We show how to use the Kron reduction method for kinetic models described in the previous section, as a tool for estimating the parameters involved in the corresponding mathematical model from time-series partial experimental data of species’ concentrations.

### 4.1. Problem Statement: Available Experimental Data

Typically, biological experiments provide measurements of species’ concentrations or reaction rates. In this manuscript, we assume that in an experimental setup some of the species’ concentrations are measured. Suppose that the output of a biological experiment corresponding to the mathematical model (8) is of the form:

z=Hx,

where $H\in R^{n\times s}$ is a constant matrix known as the *measurement matrix*. In general, *H* can have an arbitrary structure. However, in general only some of the species’ concentrations are measured experimentally. Thus, we assume that each row of *H* has only one non-zero element, which is equal to one and is placed in the position corresponding to the particular species. For instance, with reference to the CRN (4), if the species that are measured experimentally are $X_{1}$, $X_{2}$, $X_{4}$ and $X_{5}$, then

(14) $$H=\left[\begin{array}{ccccc} 1 & 0 & 0 & 0 & 0\\ 0 & 1 & 0 & 0 & 0\\ 0 & 0 & 0 & 1 & 0\\ 0 & 0 & 0 & 0 & 1 \end{array}\right].$$

Further assume that we have *l* different sets of time-series data of *z* collected from biological experiments on the same CRN. For j=1,…,l; i=1,…,n and $m=0,\ldots ,N_{j}$, let ${\hat{z}}_{i,m}^{\left(j\right)}$ be the observed value of the *i*th output at time instant $t_{m}^{\left(j\right)}$ corresponding to the *j*th experiment. For compactness, we consider the following observed experimental time-series data sets:

(15) $$\Lambda^{\left(j\right)}=\left\{\left(t_{m}^{\left(j\right)},{\hat{z}}_{i,m}^{\left(j\right)}\right)|i=1,\ldots ,n;m=0,\ldots ,N_{j}\right\},\;j=1,\ldots ,l.$$

We aim to identify the best-fitting parameters corresponding to the mathematical model (8) of the considered CRN from observed time-series data $\Lambda^{\left(j\right)}$, j=1,…,l, collected from biological experiments.

### 4.2. Trajectory-Independent Error

We propose a trajectory-independent alternative method to the computation of the error integral defined in [29] for comparing the dynamics of the Kron-reduced model to the one of the original mathematical model. In our parameter estimation procedure, this error will be used as an objective function for minimization. It is based on matching the so called relaxation constant of the Kron-reduced mathematical model to the one of the original mathematical model. We define the *relaxation constant* τ(L) of a CRN as the smallest non-zero real part of the eigenvalues of its Laplacian matrix, i.e.,

$$\tau \left(L\right)=\min_{\begin{array}{c} \lambda \in \sigma (L)\\ \lambda \neq 0 \end{array}}ℜ\left(\lambda \right).$$

The quantity τ(L) illustrates a key characteristic property of the CRN that is represented by the Laplacian matrix *L*. In the case when each complex is a single-species complex, i.e., $Z=I_{s}$, it can be shown that relaxation constant τ(L) is an exact indicator of the settling-time of the CRN, i.e., the time instant after which the concentrations of all the species fall and remain within some specified percentage of their corresponding steady-state values. This case is encountered commonly as in one of our demonstrative real-life examples considered in the next section. In this case, the solution to the balance laws (8) is of the form:

(16) $$x\left(t\right)=\sum_{i=1}^{q}\sum_{j=0}^{p_{i}-1}t^{j}w^{\left(ij\right)}\left(t\right)e^{-ℜ(\lambda_{i})t},$$

where $p_{i}$ is the multiplicity of the eigenvalue $\lambda_{i}$ and

$$w^{\left(ij\right)}=b^{\left(ij\right)}\cos\left(ℑ(\lambda_{i})t\right)+c^{\left(ij\right)}\sin\left(ℑ(\lambda_{i})t\right)\;\mathrm{with}\;a,b^{\left(ij\right)},c^{\left(ij\right)}\in R^{s}.$$

Since the real parts of eigenvalues of the Laplacian matrix *L* are all nonnegative, from (16) it follows that the settling-time of the CRN is determined by the slowest decaying term within the summation sign in the right hand side of the equation. Observe that for every i=1,…,q the terms $t^{j}w^{\left(ij\right)}\left(t\right)e^{-ℜ(\lambda_{i})t}$, $j=1,\ldots ,p_{i}-1$, decay faster than the term $w^{\left(i0\right)}\left(t\right)e^{-ℜ(\lambda_{i})t}$ whose time constant is equal to $ℜ{\left(\lambda_{i}\right)}^{-1}$. Among the terms $w^{\left(i0\right)}\left(t\right)e^{-ℜ(\lambda_{i})t}$, the slowest decaying term is the one with the biggest time constant, i.e., the one with the smallest value of $ℜ(\lambda_{i})$. Thus, for comparing the settling-time of the Kron-reduced model with the one of the original model it is reasonable to compare the relaxation constant $\tau (\hat{L})$ of the Kron-reduced model with the relaxation constant τ(L) of the original mathematical model. In general, irrespective of whether the complex composition matrix *Z* is the identity matrix or not, we quantify the difference between the dynamics of the original mathematical model and the one of the Kron-reduced model using the trajectory-independent spectral based error $\delta :R_{+}^{r}\to R_{+}$ defined as:

(17) $$\delta \left(k\right)=\left|\tau \left(L\right)-\tau \left(\hat{L}\right)\right|.$$

Note that the spectral based error δ defined in (17) is a function that only depends on the vector of parameters *k* contained in the original model. This is due to the fact that the entries as well as the eigenvalues of both the original Laplacian matrix *L* and the Kron-reduced Laplacian matrix $\hat{L}$ are functions of the parameter vector *k*.

**Remark** **1.** *In the physical sciences, the term “relaxation" usually refers to the return of a system to equilibrium. Each relaxation process can be categorized by a relaxation time (see, e.g., [47,48,49]). Since the quantity τ(L) is an indicator of the settling time of the CRN, i.e., its relaxation, we refer to the constant τ(L) as the relaxation constant of the CRN described by the Laplacian matrix L.*

### 4.3. Estimation Procedure

We turn our attention to the parameter estimation procedure. We assume that in an experimental setup certain measurements $\Lambda^{\left(j\right)}$, j=1,…,l, of the form given in (15) are collected. The principal goal is to find estimates for the parameters *k* of the original model (8) such that the corresponding model-predicted values fit the available time-series partial experimental data (15).

As explained in Section 2.5, since we do not have complete data on species’ concentrations, direct estimation of the parameters involved in the balance laws (8) is not possible. In order to convert the problem to a well-posed parameter estimation problem, we first derive the Kron-reduced mathematical model obtained after deleting a certain subset of complexes from the corresponding graph of complexes. For this purpose, we identify this subset of complexes by making use of the complex composition matrix *Z* and the measurement matrix *H*. The latter specifies the set of indices J corresponding to the species that are measured experimentally. For instance, if the measurement matrix *H* is the one given in (14), then the above-mentioned set of indices is simply $J=\left\{1,2,4,5\right\}$, i.e., only the species $X_{i}$, i=1,2,4,5, are measured experimentally. Furthermore, the complex composition matrix *Z* identifies the set of complexes $\bar{I}$ that have at least one unmeasured species. We now delete the complexes corresponding to the set of indices $\bar{I}$ using the Kron reduction method. The $\hat{s}\times \hat{c}$ complex composition matrix $\hat{Z}$ and the $\hat{c}\times \hat{r}$ incidence matrix $\hat{B}$ are computed using the procedure explained earlier.

Subsequently, we determine the parameter dependence function $f:R_{+}^{r}\to R_{+}^{\hat{r}}$. In order to express the vector of parameters $p\in R_{+}^{\hat{r}}$ of the Kron-reduced model as a function of the vector of parameters $k\in R_{+}^{r}$ of the original model, i.e p=f(k). In general, the explicit form of this function is complicated and its manual derivation is not straightforward. However, the usage of MATLAB symbolic variables allows us to derive the explicit form of the parameter dependence function *f*. Using the properties of the Kron reduction method for CRNs governed by mass action kinetics rate law, we conclude that the dependence function is a vector-valued rational function of its argument.

We now consider the parameter estimation problem for the Kron-reduced mathematical model from the available observed time-series experimental data of species’ concentrations (15). It is a well-posed parameter estimation problem since the available experimental data corresponds to all the concentrations involved in the Kron-reduced mathematical model. We apply the least squares optimisation technique to determine the best-fitting values of parameters $\hat{p}\in R^{\hat{r}}$, i.e., the parameter values for which the available observed time-series experimental data $\Lambda^{\left(j\right)}$, j=1,…,l given in (15) fits the Kron-reduced model.

Finally, we determine the values of parameters $\hat{k}\in R^{r}$ for which the trajectory-independent spectral-based error function δ given in (17) admits its minimum value subject to the constraint

(18) $$f\left(k\right)=\hat{p}.$$

In other words, we have the following well-posed constrained optimisation problem

$$\min_{k\in R_{+}^{r}}\delta \left(k\right)\;\mathrm{subject}\;\mathrm{to}\;f\left(k\right)=\hat{p}.$$

We use the method of Lagrange multipliers to solve the above-mentioned problem using MATLAB Optimization Toolbox. We developed a MATLAB library for the automation of our estimation procedure. The inputs required for our parameter estimation procedure are the s×c complex composition matrix *Z*, the c×r incidence matrix *B*, the n×s measurement matrix *H*, and the observed time-series experimental datasets $\Lambda^{\left(j\right)}$, j=1,…,l. The output of the parameter estimation method is the vector $\hat{k}\in R_{+}^{r}$ of the best-fitting parameter values corresponding to the experimental time-series observed data given in (15).

## 5. Application to Real-Life Examples

We demonstrate the applicability of our automated parameter estimation algorithm from observed time-series partial experimental data of species’ concentrations on two real-life computational models of biological processes retrieved from the BioModels database [32]. We consider a model of nicotinic acetylcholine receptors [33] and a model of Trypanosoma brucei trypanothione synthetase [34]. For each of these models, we first generate partial time-series data corresponding to the species’ concentrations using the values of parameters provided in the corresponding reference. Next, we perturb these generated data with white Gaussian noise with zero mean and sufficiently small standard deviation. We then apply our estimation technique to determine the best-fitting values of parameters in a fully automated manner. The corresponding MATLAB library is provided as Supplementary Material.

### 5.1. Nicotinic Acetylcholine Receptors

**Figure 1:**
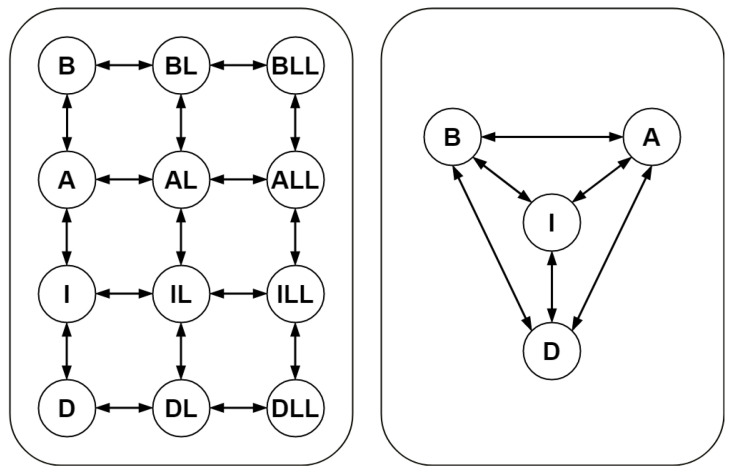
Schematic representation of the original model (**left-hand panel**) of nicotinic acetylcholine receptors and the corresponding Kron-reduced model (**right-hand panel**) obtained by deleting the single-species complexes BL, AL, IL, DL, BLL, ALL, ILL, and DLL from the graph of complexes.

We consider a model of nicotinic acetylcholine receptors (NAR) developed in [33]. Nicotinic receptors are receptor polypeptides (short chains of amino acids) that respond to the neurotransmitter acetylcholine (a signalling molecule secreted by a neuron) as well as to drugs such as the agonist nicotine. They are found in the central and peripheral nervous systems, muscles, and several other tissues of different organisms. A schematic representation of the network corresponding to the mathematical model of NAR is provided in the left-hand panel of Figure 1. A detailed description of the mathematical model can be found in [33].

#### 5.1.1. The Considered Model and the Available Data

In the considered model of NAR, there are 12 chemical species participating in 17 reversible mass action reactions. All complexes here are single-species complexes. There are thus 12 complexes in the corresponding graph of complexes. As mentioned earlier, for the purpose of automated modelling purposes we regard each reversible reaction as two separate unidirectional reactions. Table 1 provides an overview of the primary compounds participating in the reaction system.

The rest of the compounds are intermediate enzyme complexes participating in the reaction network. The network consists of the following reactions, all of which are reversible.

$B\;\;\overset{k_{1}}{\underset{k_{2}}{⇌}}\mathrm{BL}$,  $B\;\;\overset{k_{11}}{\underset{k_{12}}{⇌}}A$,$\mathrm{BL}\;\;\overset{k_{3}}{\underset{k_{4}}{⇌}}\;\;\mathrm{BLL}$,  $\mathrm{BL}\;\;\overset{k_{13}}{\underset{k_{14}}{⇌}}\;\;\mathrm{AL}$,$\mathrm{BLL}\;\;\overset{k_{5}}{\underset{k_{6}}{⇌}}\;\;\mathrm{ALL}$,  $I\;\;\overset{k_{15}}{\underset{k_{16}}{⇌}}\;\;\mathrm{IL}$,$A\;\;\overset{k_{7}}{\underset{k_{8}}{⇌}}\mathrm{AL}$,  $\mathrm{IL}\;\;\overset{k_{17}}{\underset{k_{18}}{⇌}}\;\;\mathrm{ILL}$,$\mathrm{AL}\;\;\overset{k_{9}}{\underset{k_{10}}{⇌}}\;\;\mathrm{ALL}$,  $A\;\;\overset{k_{19}}{\underset{k_{20}}{⇌}}\;\;I$,$\mathrm{AL}\;\;\overset{k_{21}}{\underset{k_{22}}{⇌}}\;\;\mathrm{IL}$,  $I\;\;\overset{k_{29}}{\underset{k_{30}}{⇌}}\;\;D$,$\mathrm{ALL}\;\;\overset{k_{23}}{\underset{k_{24}}{⇌}}\;\;\mathrm{ILL}$,  $\mathrm{IL}\;\;\overset{k_{31}}{\underset{k_{32}}{⇌}}\;\;\mathrm{DL}$,$D\;\;\overset{k_{25}}{\underset{k_{26}}{⇌}}\;\;\mathrm{DL}$,  $\mathrm{ILL}\;\;\overset{k_{33}}{\underset{k_{34}}{⇌}}\;\;\mathrm{DLL}$.$\mathrm{DL}\;\;\overset{k_{27}}{\underset{k_{28}}{⇌}}\;\;\mathrm{DLL}$,
The values of the parameters $k_{i}$, i=1,…,34, provided in [33] are given in Table 2. We assume that the species that are measured in an experimental setup are the ones having a greater impact on the reactions, which are the activatable resting closed state B (the Basal state), the state of higher affinities with open channel A (the Active state), the states of higher affinities with closed channels I (the Inactivatable state) and D (the Desensitized state). In other words, the corresponding measurement matrix is:

$$\begin{array}{c} H=\left[\begin{array}{cc} I_{4} & {𝟘}_{4\times 8} \end{array}\right]. \end{array}$$

We generate data for these species using the values of parameters provided in [33]. Additionally, we perturb the obtained data with white Gaussian noise of zero mean and standard deviation 0.0001. We explain how to estimate the parameters involved in the corresponding mathematical model from the above-mentioned time-series partial data of concentrations using our parameter estimation method.

#### 5.1.2. Proving Parameter Unidentifiability

Before we perform the parameter estimation, we show that the parameters of the mathematical model under consideration cannot be uniquely determined from the available partial time series data of the species’ concentrations. The reason is that we are able to provide two different vectors of parameters, both leading to the same system of ODEs corresponding to the measured concentrations of the species. Consider two vectors of parameters $k,\bar{k}\in R_{+}^{34}$ with the following properties:

$$\begin{array}{ccc} k_{i} & = & {\bar{k}}_{i},\;\;\;i=1,7,11,12,15,19,20,25,29,30,\\ k_{i} & = & {\bar{k}}_{i}=0,\;\;i=2,8,16,26,\\ k_{i} & \neq & {\bar{k}}_{i},\;\;\;\mathrm{otherwise}. \end{array}$$

Any two vectors of parameters *k* and $\bar{k}$ with these properties lead to the same ODE system corresponding to the measured concentrations of the species, and thus to the same sets of admissible output trajectories. Note that it may be cumbersome to derive these properties manually. Therefore, we have used MATLAB symbolic variables to derive these properties in an automatic way. We conclude that the corresponding mathematical model is parameter unidentifiable. From Theorem 2 it follows that the considered mathematical model is also least squares parameter unidentifiable.

#### 5.1.3. Parameter Estimation Procedure

According to our procedure, we first determine the Kron reduced mathematical model obtained by deleting the complexes involving at least one species that is not measured experimentally. By making use of the complex composition matrix and the measurement matrix we identify the single-species complexes BL, AL, IL, DL, BLL, ALL, ILL, and DLL to be the ones that should be deleted from the original mathematical model. These are the precisely the species whose concentrations are not measured. We consequently obtain a new mathematical model in which only the concentrations of the measured species are involved. The reactions corresponding to the Kron reduced mathematical model are given below:

$B\;\;\overset{p_{1}}{\underset{p_{2}}{⇌}}\;\;A$,  $I\;\;\overset{p_{5}}{\underset{p_{6}}{⇌}}D$,  $B\;\;\overset{p_{9}}{\underset{p_{10}}{⇌}}\;\;D$,$A\;\;\overset{p_{3}}{\underset{p_{4}}{⇌}}I$,  $A\;\;\overset{p_{7}}{\underset{p_{8}}{⇌}}\;\;D$,  $B\;\;\overset{p_{11}}{\underset{p_{12}}{⇌}}\;\;I$.

The Kron reduced network is schematically illustrated in the right-hand panel of Figure 1. Recall that the vector of parameters *p* is a function of the vector of parameters *k*. We now possess a complete time-series data of species’ concentrations corresponding to the Kron reduced mathematical model. In other words, we have a well-posed parameter estimation problem from species’ concentrations, which is solved using the well known least squares method. In order to test the performance of our parameter estimation method, we use both UWLS and WLS to determine the corresponding values of the parameters for which the Kron reduced mathematical model fits the available data of species’ concentrations. These estimates are given in Table 3.

**Figure 2:**
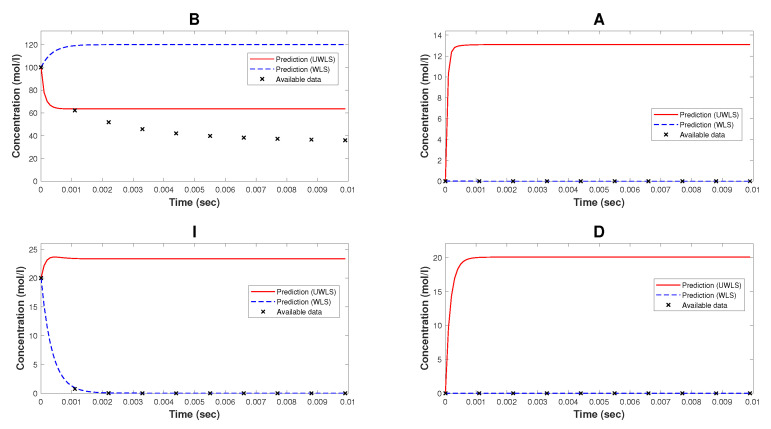
The available time-series data of species’ concentrations of the model of nicotinic acetylcholine receptors and the corresponding predicted values (using WLS and UWLS) obtained from the Kron reduced mathematical model with estimated parameters provided in Table 3.

Figure 2 represents the comparison of the available time-series data of species’ concentrations of the model of NAR and the corresponding predicted values (for both WLS and UWLS approaches) obtained from the Kron reduced mathematical model with estimated parameters provided in Table 3.

**Figure 3:**
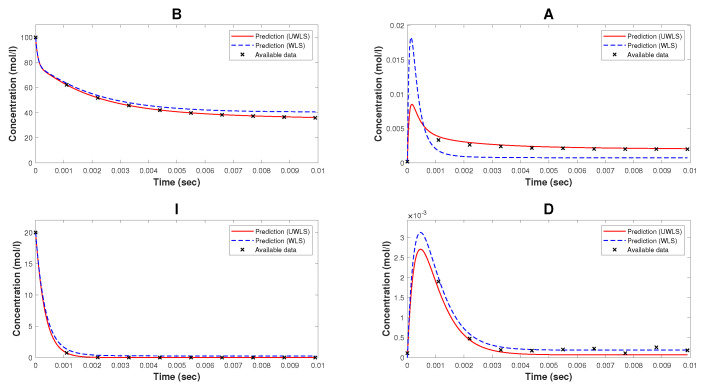
The available time-series data of species’ concentrations of the model of nicotinic acetylcholine receptors and the corresponding model predicted values (for both WLS and UWLS approaches) with parameters estimated by our method. These estimated parameters are given in Table 2.

Note that, while Kron reduction method effectively reduces the complexity of the original mathematical model, it may not fully capture all relevant dynamical properties. As can be seen from Figure 2, the Kron reduced mathematical model is not a good fit for the available time-series data. Finally, we determine the values of parameters $\hat{k}$ (for both WLS and UWLS) that minimize the eigenvalue-based error δ defined in (17) subject to the constraint defined in (18). These best-fitting values of parameters are provided in Table 2. The comparison of the available time-series data of species’ concentrations and the corresponding model predicted values obtained from (8) with estimated parameter values $\hat{k}$ (for both WLS and UWLS approaches) is given in Figure 3.

We observe that our parameter estimation method is able to derive a complete mathematical model that is able to make accurate predictions about the dynamics of the CRN. Note from Table 2 that the estimated values of some of the parameters, e.g., $k_{i}$, i=21,…,24 and i=31,…,34, differ by a large percentage from their corresponding values provided in [33]. However, the mathematical model with the estimated values of parameters is a reasonably good fit (as can be seen in Figure 3) for the generated time-series data of species’ concentrations. The reason behind this, as discussed earlier, is the fact that the parameters involved in the considered mathematical model of NAR are not least squares identifiable from the output corresponding to the compounds B, A, I, and D, as proved in Section 5.1.2.

Recall that in our parameter estimation method, we make use of the least squares optimization technique. As mentioned above, we used both WLS and UWLS to determine the best-fitting values of parameters corresponding to each of these approaches (see Table 2). We perform LOOCV in order to understand which one of these approaches is a preferable technique for data-fitting in our proposed parameter estimation procedure. We first give a short summary of LOOCV. It involves splitting the data set into two parts: a single datapoint, that is used for validation (excluded datapoint); and the remaining datapoints (training dataset), that make up the set used for parameter estimation. The parameter estimation procedure is applied using the training dataset, and a model-predicted value is computed for the excluded datapoint. We then compute the mean squared error between the excluded datapoint and its corresponding model-predicted values. Since the excluded datapoint is not used in the fitting process, this error provides an unbiased estimate for the test error. On the other hand, it is a poor estimate for the test error since it is based upon a single datapoint. We can repeat the procedure by excluding each datapoint of the given data exactly once and compute the mean squared error between the excluded datapoint and its corresponding model-predicted values. The LOOCV training error is the average of these test error estimates.

We used WLS in our parameter estimation procedure and computed the LOOCV training error. Subsequently, we used UWLS in our parameter estimation procedure and again computed the LOOCV training error. These training errors are provided in Table 4.

As we can see from this table, using UWLS in our parameter estimation procedure results in a smaller LOOCV training error than using WLS. We thus conclude that, for this particular dataset, UWLS is a preferable approach of data-fitting in our proposed parameter estimation procedure.

We calculated the 95% confidence intervals for the estimated values of parameters corresponding to UWLS by performing bootstrapping (see, e.g., [50,51,52,53]) for our parameter estimation method. The procedure for this calculation is as follows. We first construct 2000 resampled datasets (of the same length as the available data) by randomly choosing datapoints from the available data. Note that the same datapoint can be chosen multiple times. Secondly, we use our parameter estimation method to determine the best-fitting values of parameters corresponding to each of the resampled dataset. As a result, for each of the parameters we obtain a set of 2000 estimates. The 95% bootstrap confidence intervals were constructed by choosing 2.5% and 97.5% percentiles of the corresponding bootstrap estimates. These confidence intervals are included in Table 2.

### 5.2. Trypanosoma Brucei Trypanothione Synthetase

**Figure 4:**
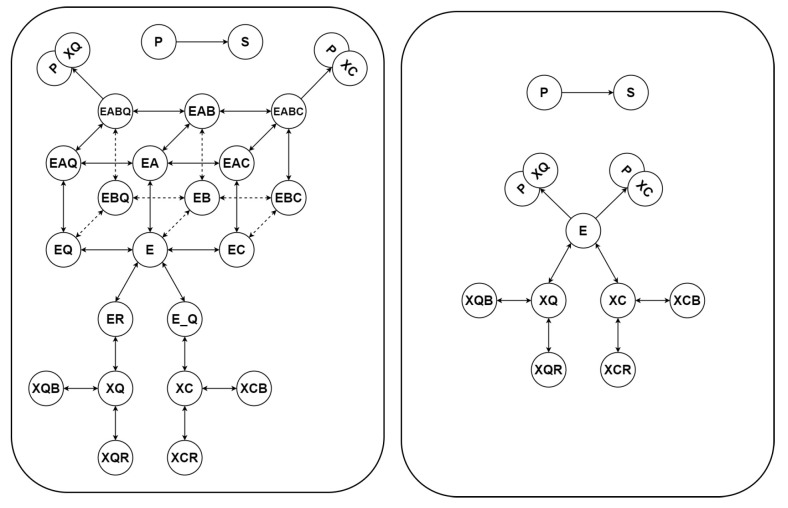
Schematic representation of the original model (**left-hand panel**) of trypanosoma brucei trypanothione synthetase and the corresponding Kron-reduced model (**right-hand panel**) obtained by deleting the single-species complexes EA, EB, EC, EQ, ER, E_Q, EAB, EAC, EAQ, EBC, EBQ, EABQ, EABC from the graph of complexes.

Subsequently, we demonstrate the applicability of our new parameter estimation method on a very different type of a CRN. We consider a kinetic model of Trypanosoma brucei trypanothione synthetase (TBTS). This mathematical model of TBTS was developed in [34] and describes the entire kinetic profile. Trypanosoma brucei is a species of parasitic kinetoplastid (an organism whose cells contain a cell nucleus and that is not an animal, plant, or fungus). Unlike other parasites (normally infecting blood and tissue cells) it is exclusively extracellular and inhabits the blood plasma as well as body fluids. It causes deadly diseases in humans such as African trypanosomiasis or sleeping sickness. A trypanothione synthetase is a catalytic enzyme. A schematic representation of the model of TBTS is shown on the left-hand side of Figure 4.

#### 5.2.1. The Considered Model and the Available Data

In the considered mathematical model of TBTS, there are 22 species participating in 59 unidirectional reactions in terms of 24 distinct complexes. Table 5 provides an overview of the primary compounds participating in the reaction system.

The remaining compounds occurring in the network are intermediate complexes of the enzymes E and X. The reactions occurring in the network are given as:
$E\;\;\overset{k_{1}}{\underset{k_{2}}{⇌}}\;\;\mathrm{EA}$,  $\mathrm{XQ}\;\;\overset{k_{30}}{\underset{k_{31}}{⇌}}\;\;\mathrm{XQB}$,$\mathrm{EA}\;\;\overset{k_{3}}{\underset{k_{4}}{⇌}}\;\;\mathrm{EAB}$,  $\mathrm{XQ}\;\;\overset{k_{32}}{\underset{k_{33}}{⇌}}\;\;\mathrm{XQR}$,$E\;\;\overset{k_{5}}{\underset{k_{6}}{⇌}}\;\;\mathrm{EB}$,  $E\;\;\overset{k_{34}}{\underset{k_{35}}{⇌}}\;\;\mathrm{EC}$,$\mathrm{EB}\;\;\overset{k_{7}}{\underset{k_{8}}{⇌}}\;\;\mathrm{EAB}$,  $\mathrm{EC}\;\;\overset{k_{36}}{\underset{k_{37}}{⇌}}\;\;\mathrm{EAC}$,$\mathrm{EAB}\;\;\overset{k_{9}}{\underset{k_{10}}{⇌}}\;\;\mathrm{EABQ}$,  $\mathrm{EAC}\;\;\overset{k_{38}}{\underset{k_{39}}{⇌}}\;\;\mathrm{EABC}$,$\mathrm{EA}\;\;\overset{k_{11}}{\underset{k_{12}}{⇌}}\;\;\mathrm{EAQ}$,  $\mathrm{EC}\;\;\overset{k_{40}}{\underset{k_{41}}{⇌}}\;\;\mathrm{EBC}$,$\mathrm{EB}\;\;\overset{k_{13}}{\underset{k_{14}}{⇌}}\;\;\mathrm{EBQ}$,  $\mathrm{EBC}\;\;\overset{k_{42}}{\underset{k_{43}}{⇌}}\;\;\mathrm{EABC}$,$E\;\;\overset{k_{15}}{\underset{k_{16}}{⇌}}\;\;\mathrm{EQ}$,  $\mathrm{EA}\;\;\overset{k_{44}}{\underset{k_{45}}{⇌}}\;\;\mathrm{EAC}$,$\mathrm{EQ}\;\;\overset{k_{17}}{\underset{k_{18}}{⇌}}\;\;\mathrm{EAQ}$,  $\mathrm{EB}\;\;\overset{k_{46}}{\underset{k_{47}}{⇌}}\;\;\mathrm{EBC}$,$\mathrm{EAQ}\;\;\overset{k_{19}}{\underset{k_{20}}{⇌}}\;\;\mathrm{EABQ}$,  $\mathrm{EAB}\;\;\overset{k_{48}}{\underset{k_{49}}{⇌}}\;\;\mathrm{EABC}$,$\mathrm{EQ}\;\;\overset{k_{21}}{\underset{k_{22}}{⇌}}\;\;\mathrm{EBQ}$,  $\mathrm{EABC}\;\;\overset{k_{50}}{\to }\;\;P+\mathrm{XC}$,$\mathrm{EBQ}\;\;\overset{k_{23}}{\underset{k_{24}}{⇌}}\;\;\mathrm{EABQ}$,  $\mathrm{XC}\;\;\overset{k_{51}}{\underset{k_{52}}{⇌}}\;\;E\_Q$,$\mathrm{EABQ}\;\;\overset{k_{25}}{⇌}\;\;P+\mathrm{XQ}$,  $E\_Q\;\;\overset{k_{53}}{\underset{k_{54}}{⇌}}\;\;E$,$\mathrm{XQ}\;\;\overset{k_{26}}{\underset{k_{27}}{⇌}}\;\;\mathrm{ER}$,  $\mathrm{XC}\;\;\overset{k_{55}}{\underset{k_{56}}{⇌}}\;\;\mathrm{XCB}$,$\mathrm{ER}\;\;\overset{k_{28}}{\underset{k_{29}}{⇌}}\;\;E$,  $\mathrm{XC}\;\;\overset{k_{57}}{\underset{k_{58}}{⇌}}\;\;\mathrm{XCR}$,$P\;\;\overset{k_{59}}{\to }\;\;S$.
where $k_{i}$ is the rate constant of the *i*th reaction. The values of these parameters provided in [34] are listed in Table 6.

Note that, unlike the previous example, not every complex is a single-species complex. We assume that the species that are measured experimentally are P, S, XQ, XC, XQB, XQR, XCB, XCR and the main enzyme E. We generate data corresponding to the concentrations of the above-mentioned species using the balance laws (8) and the parameter values provided in [34]. These parameters are given in Table 6. Additionally, the obtained data have been perturbed with white Gaussian noise of zero mean and standard deviation 0.02.

#### 5.2.2. Proving Parameter Unidentifiability

We show that the parameters of the considered mathematical model cannot be uniquely determined from the available partial time series data of the species’ concentrations. Similar to the case of the mathematical model of NAR, we provide two different vectors of parameters, both leading to the same system of ODEs corresponding to the measured concentrations of the species. Consider two vectors of parameters $k,\bar{k}\in R_{+}^{59}$ with the following properties:

$$\begin{array}{ccc} k_{i} & = & {\bar{k}}_{i},\;\;i=1,2,5,6,15,16,19,25,\ldots ,35,50,\ldots ,59,\\ k_{i} & \neq & {\bar{k}}_{i},\;\;\mathrm{otherwise}. \end{array}$$

Any two vectors of parameters *k* and $\bar{k}$ with these properties lead to the same ODE system corresponding to the measured species’ concentrations, and thus to the same sets of admissible output trajectories. These properties have been derived in an automatic way using MATLAB symbolic variables. We conclude that the corresponding mathematical model is parameter unidentifiable. From Theorem 2 it follows that the considered mathematical model is also lest squares parameter unidentifiable.

#### 5.2.3. Parameter Estimation Procedure

Using the complex composition matrix and the measurement matrix our procedure selects the single-species enzymatic complexes EA, EB, EC, EQ, ER, E_Q, EAB, EAC, EAQ, EBC, EBQ, EABC, EABQ to form the set of complexes that should be deleted from the graph of complexes by applying the Kron reduction method. The reactions corresponding to the resulting Kron-reduced model are given as:

$E\;\;\overset{p_{1}}{\to }\;\;P+\mathrm{XQ}$,  $\mathrm{XC}\;\;\overset{p_{9}}{\underset{p_{10}}{⇌}}\;\;E$,$E\;\;\overset{p_{2}}{\to }\;\;P+\mathrm{XC}$,  $\mathrm{XC}\;\;\overset{p_{11}}{\underset{p_{12}}{⇌}}\;\;\mathrm{XCB}$,$\mathrm{XQ}\;\;\overset{p_{3}}{\underset{p_{4}}{⇌}}\;\;E$,  $\mathrm{XC}\;\;\overset{p_{13}}{\underset{p_{14}}{⇌}}\;\;\mathrm{XCR}$,$\mathrm{XQ}\;\;\overset{p_{5}}{\underset{p_{6}}{⇌}}\;\;\mathrm{XQB}$,  $P\;\;\overset{p_{15}}{\to }\;\;S$.$\mathrm{XQ}\;\;\overset{p_{7}}{\underset{p_{8}}{⇌}}\;\;\mathrm{XQR}$,
Here, as usual, for every i=1,…,15, $p_{i}$ denotes the rate constant of the *i*th reaction corresponding to the Kron-reduced model. The schematic representation of the Kron reduced network is illustrated in the right-hand panel of Figure 4.

**Figure 5:**
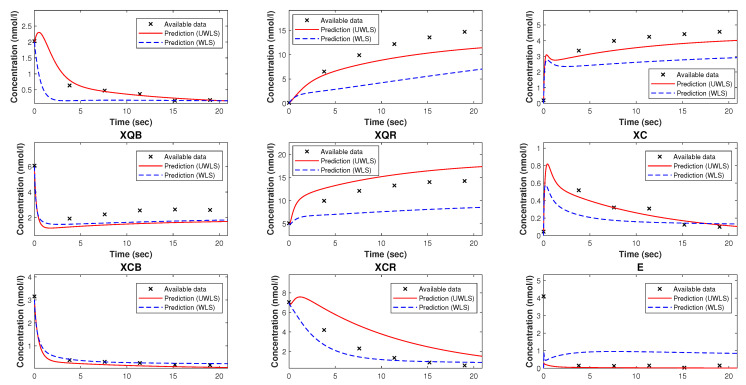
The available time-series data of species’ concentrations of the model of trypanosoma brucei trypanothione synthetase and the corresponding predicted values obtained from the Kron-reduced mathematical model with estimated parameters provided in Table 7.

Since we have complete data of the concentrations of the species involved in the Kron reduced model, we may apply the least squares method to find the best-fitting values of the parameters $\hat{p}\in R_{+}^{15}$. Similar to the case of the mathematical model of NAR, we use both WLS and UWLS to determine the values of the parameters for which the Kron reduced mathematical model fits the available data of species’ concentrations. These estimates are given in Table 7. Figure 5 visualizes the comparison of the available time-series data and the corresponding model predicted values (for both WLS and UWLS approaches) obtained from the Kron reduced model with estimated values of parameters $\hat{p}$. Note that, in this case, both WLS and UWLS result in similar model-predicted values for the output concentrations. Also note that the Kron reduced mathematical model is not a good fit for the available time-series data.

**Figure 6:**
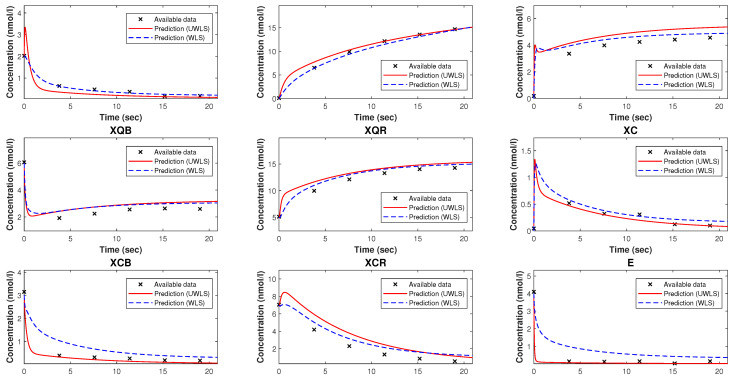
The available time-series data of species’ concentrations of the model of trypanosoma brucei trypanothione synthetase and the corresponding model predicted values with parameters estimated by our method. These estimated parameters are given in Table 6.

In the final step, we determine the values of parameters $\hat{k}\in R_{+}^{59}$ (for both WLS and UWLS approaches) of the original model that minimize the eigenvalue-based error δ defined in (17) subject to the constraint defined in (18). These values of parameters are given in Table 6. The comparison of the available time-series data and the corresponding model predicted values obtained from the balance laws (8) with the estimated values of parameters $\hat{k}$ (for both WLS and UWLS approaches) be seen in Figure 6. Note that our parameter estimation method resulted in a complete mathematical model that is able to make accurate predictions about the dynamical behavior of the CRN.

Observe from Table 6 that for some of the parameters, e.g., $k_{i}$, i=7,…,14 and i=20,…,24, there is a substantial difference between the values provided in [54] and their corresponding estimated values. The reason behind this is the fact that the parameters contained in the mathematical model are least squares unidentifiable from the available time-series partial data of species’ concentrations, as proved in Section 5.2.2.

Similar to the case of the mathematical model of NAR, we perform LOOCV to understand which one of WLS and UWLS is a more preferable approach for data-fitting in our proposed parameter estimation procedure. The corresponding LOOCV training errors are given in Table 8. As we can see from this table, unlike the case of NAR, using WLS in our parameter estimation procedure results in a smaller LOOCV training error than using UWLS. We thus conclude that, for this particular dataset, WLS is a more preferable approach of data-fitting in our proposed parameter estimation procedure. We calculate the 95% confidence intervals for the estimated values of parameters corresponding to WLS by performing bootstrapping for our parameter estimation method. These confidence intervals are included in Table 6.

## 6. Discussion

The Kron-reduced mathematical model with the best-fitting values of parameters (in the sense of least squares), as we can see from Figure 2 and Figure 5, is generally not an appropriate approximation, meaning that the corresponding model predicted values are far from being good fits for the available time-series data. This is because of the fact that, in general depending on the number of complexes deleted from the graph of complexes, it is not assured that the Kron-reduced model is a reasonable approximation for the original mathematical model.

The choice of the Kron reduction technique as a tool for reducing mathematical models in our parameter estimation method is based on several advantages of this reduction technique that are particularly appropriate for the problem. First of all, it does not impose any restrictions on the choice of complexes to be deleted. Thus, we can delete all the complexes containing at least a single unmeasured species. A second advantage is that Kron reduction method preserves the kinetics of the CRN, i.e., if MAKRL governs the given CRN, then the corresponding Kron-reduced model is also governed by this rate law. A third advantage of the Kron reduction method is that we are able to compare the dynamics of the original model to the one of the reduced model using the Laplacian matrix of the original model and the Laplacian matrix of the reduced model. To the best of our knowledge, there is no other reduction technique that offers all these aforementioned advantages.

The suggested parameter estimation method is only applicable to a mass action CRN with a constant Laplacian. This is because of the fact that in the estimation procedure, the eigenvalues of the Laplacian matrix are used. For a general enzymatic CRN, the corresponding Laplacian matrix is not constant since it depends on the vector of species’ concentrations. In such cases, it is not straightforward how to use a similar technique for parameter estimation purposes. The parameter estimation of enzyme kinetic reaction networks from partial data of species’ concentrations is still an open problem that will be considered in future work.

As explained in [37,41], a *linkage class* of a CRN is a connected component of its graph of complexes. It is also stated in these papers that if a network has a linkage class with only one reaction, then the removal of a complex involved in such a reaction by Kron reduction leads to the removal of the reaction. In the case, where the intermediate Kron reduction phase of our parameter estimation procedure leads to the removal of some of the reactions of the original model, we would expect that the estimated parameters of the original model associated with the removed reactions would have larger confidence intervals compared with those of the other parameters that are associated with the remaining reactions of the network.

## 7. Conclusions

In this paper, we have introduced an innovative parameter estimation approach for mathematical models of mass action CRNs using observed time-series incomplete experimental data of species’ concentrations. As far as we know, there exists no direct technique for deducing the parameters in a mathematical model from this sort of experimental data. We have addressed this problem by devising an algorithmic strategy, which involves the application of Kron reduction technique for kinetic models as an intermediate step in the overall parameter estimation approach. The complexes that should be deleted using Kron reduction are chosen in such a way that in the reduced model only the concentrations of the measured species are involved. Since all the species’ concentrations involved in the Kron-reduced model are measured we now have a well-posed parameter estimation problem. We estimate the parameters involved in the Kron-reduced model using the least squares method to identify the best-fitting values of the parameters involved in the Kron-reduced model. To estimate the parameters contained in the original mathematical model, we have devised a new trajectory-independent measure to quantify the difference between the dynamics of the original model and the corresponding Kron-reduced model. It is based on comparing the smallest non-zero real part of the eigenvalues of the original Laplacian matrix with the one of the Kron-reduced Laplacian matrix. The reason behind the choice of measure is the fact that the smallest non-zero real part of the Laplacian matrix is related to the settling time of the CRN that is characterized by the Laplacian matrix. This measure can be regarded as a function of the parameter vector of the original mathematical model since the eigenvalues of both the original Laplacian matrix as well as the Kron-reduced Laplacian matrix depend only on this vector of parameters. Finally, we find the estimates of the parameters for which the above-mentioned spectral-based measure admits its smallest value.

We have devised an automatic process for our parameter estimation approach, crafting a MATLAB library that can be employed to deduce the parameters from experimental data of species’ concentrations automatically. This MATLAB library is given as Supplementary Material. We utilized this MATLAB library to effectively employ our parameter estimation approach on two real-life CRNs taken from the Biomodels database [32]. For each of these models, we observed that our parameter estimation method resulted in a complete mathematical model that could make accurate predictions about the dynamics of the CRN. While we have exclusively applied and tested our parameter estimation method on a limited scale, involving only two real-life instances of CRNs, its applicability extends to all networks regulated by MAKRL. It should be noted that the method places no restriction on the size or scale of the model as well as the biological purpose of the associated network as long as its reactions are governed by MAKRL. Thus the method is applicable for models of core metabolism (for instance E.coli central carbon metabolism models reviewed in [55]) as well as models of regulatory networks. It can also be applied for small models like the one of NAR considered in this paper as well as genome-scaled models as in [56].

## Figures and Tables

**Table 1:** An overview of the primary compounds participating in the considered model of the nicotinic acetylcholine receptors.

Species Notation	Species Name	Species ID
A	State of higher affinities with open channel	Active
B	Activatable resting closed state	Basal
I	Inactivatable state of higher affinities with closed channel	Inactivatable
D	Desensitised state of higher affinities with closed channel	Desensitised

**Table 2:** The values of the rate constants of the model of the nicotinic acetylcholine receptors provided in [33], the estimated values obtained using our method (for both WLS and UWLS), and the corresponding confidence intervals calculated using bootstrapping.

Parameters	Provided Values	Estimated Values (UWLS)	Estimated Values (WLS)	Confidence Intervals
$k_{1}$	3000.0000	2985.3982	2999.1102	[2975.0001, 3004.0154]
$k_{2}$	8000.0000	7997.6479	7992.4287	[7658.0406, 8106.2202]
$k_{3}$	1500.0000	1499.4586	1486.9596	[1400.0112, 1587.4735]
$k_{4}$	16,000.00	15,995.28	15,978.15	[15,116.77, 16,007.37]
$k_{5}$	30,000.0000	29,991.0951	29,956.5732	[2821.5981, 3033.6088]
$k_{6}$	700.0000	700.4901	722.1788	[675.6376, 896.1618]
$k_{7}$	3000.0000	3011.9891	3038.551	[2999.0560, 3066.3532]
$k_{8}$	8.6400	2.0077	36.7439	[1.4659, 237.2756]
$k_{9}$	1500.0000	1499.3846	1496.7745	[1424.2376, 1608.9460]
$k_{10}$	17.2800	18.0771	5.4371	[2.3910, 26.9894]
$k_{11}$	0.5400	0.7522	0.0934	[$2.82\times 10^{-5}$, 0.7812]
$k_{12}$	10,800.00	10,809.95	10,822.44	[10,800.01, 10,833.63]
$k_{13}$	130.0000	129.8977	0.01295	[0.0002, 145.5059]
$k_{14}$	2740.0000	2739.3315	2736.4242	[2546.3118, 2843.7101]
$k_{15}$	3000.0000	3000.5636	2848.1724	[2772.8053, 3000.0009]
$k_{16}$	4.0000	3.2436	162.0834	[1.1588, 211.1424]
$k_{17}$	1500.0000	1500.0776	1491.0929	[1488.1059, 1513.4998]
$k_{18}$	8.0000	11.4275	378.6929	[6.9902, 503.0242]
$k_{19}$	19.7000	19.6393	0.6956	[0.1307, 12.2759]
$k_{20}$	3.7400	4.0754	5.5581	[1.8582, 27.4541]
$k_{21}$	19.8500	18.9582	104.6638	[17.6869, 190.2117]
$k_{22}$	1.7400	0.2558	66.9278	[0.0327, 77.2369]
$k_{23}$	20.0000	17.8055	0.0246	[0.0014, 23.0991]
$k_{24}$	0.8100	0.0019	0.01142	[0.8588, 2.6275]
$k_{25}$	3000.0000	2999.178	2998.2315	[2880.6353, 3012.3044]
$k_{26}$	4.0000	4.0298	4.4320	[1.4111, 5.6222]
$k_{27}$	1500.0000	1499.6145	1500.2207	[960.5602, 1739.9138]
$k_{28}$	8.0000	5.7722	14.2218	[3.6051, 18.2161]
$k_{29}$	0.0500	0.0041	0.0566	[$3.93\times 10^{-6}$, 0.0567]
$k_{30}$	0.0010	0.3051	0.4618	[0.0002, 6.9397]
$k_{31}$	0.0500	$4.99\times 10^{-6}$	0.0015	[$5.15\times 10^{-7}$, 68.9054]
$k_{32}$	0.0010	0.0006	0.0577	[0.0001, 1.8794]
$k_{33}$	0.0500	$6.78\times 10^{-8}$	0.0028	[$1.3\times 10^{-8}$, 0.5321]
$k_{34}$	0.0010	$1.60\times 10^{-6}$	0.0009	[$0.85\times 10^{-6}$, 0.0021]

**Table 3:** The best-fitting parameter values (for both WLS and UWLS approaches) of the Kron reduced mathematical model of the nicotinic acetylcholine receptors corresponding to the available time-series experimental data.

Parameters	Estimated Values (UWLS)	Estimated Values (WLS)
$p_{1}$	2060.8744	0.2430
$p_{2}$	947.0466	0.6338
$p_{3}$	1385.3214	$1.73\times 10^{-5}$
$p_{4}$	13,732.9647	13,783.3992
$p_{5}$	1061.5483	53.9256
$p_{6}$	8.6512	1.1069
$p_{7}$	2860.8880	2762.7458
$p_{8}$	313.8923	16.0378
$p_{9}$	0.0192	0.2593
$p_{10}$	1617.0009	340.8659
$p_{11}$	2769.5029	3.8327
$p_{12}$	1.3036	88.8853

**Table 4:** Leave-one-out cross-validation training errors for the mathematical model of the nicotinic acetylcholine receptors corresponding to weighted and unweighted least squares optimization methods.

	Unweighted Least Squares	Weighted Least Squares
Training error	3.2164	3.6108

**Table 5:** An overview of the primary compounds participating in the considered model of the Trypanosoma brucei trypanothione synthetase.

Species Notation	Species Name	Species ID
E	Enzyme	E
P	Adenosine diphosphate	ADP
Q	Glutathionylspermidine	GSP
R	Bis(glutathionyl)spermine	${T(\mathrm{SH})}_{2}$
C	Spermidine	Spd
B	Glutathione	GSH
A	Adenosine triphosphate	ATP
X	Glutathione spermidine Enzyme	E.GS_P

**Table 6:** The values of the rate constants of the model of the trypanosoma brucei trypanothione synthetase provided in [34], the estimated values obtained using our method (for both WLS and UWLS), and the corresponding confidence intervals calculated using bootstrapping.

Parameters	Provided Values	Estimated Values (UWLS)	Estimated Values (WLS)	Confidence Intervals
$k_{1}$	53.4178	44.1002	42.8180	[36.3628, 53.1468]
$k_{2}$	9.06800	16.7531	12.4590	[8.4905, 15.9902]
$k_{3}$	1.1917	8.7110	3.9547	[1.6893, 7.7521]
$k_{4}$	3.6300	16.4578	4.8968	[3.7351, 7.9581]
$k_{5}$	67.8700	58.5546	68.7404	[51.6995, 69.8990]
$k_{6}$	9.5200	62.8531	0.3219	[0.0410, 10.7708]
$k_{7}$	7.9700	13.8148	0.2034	[0.0662, 6.8344]
$k_{8}$	4.5000	52.2816	5.1613	[4.6057, 10.0443]
$k_{9}$	1.7600	1.8273	5.2016	[0.0798, 7.7487]
$k_{10}$	5.5000	11.7959	11.5207	[5.9256, 13.4458]
$k_{11}$	5.0734	85.8926	0.5021	[0.0919, 8.6912]
$k_{12}$	5.0396	54.9415	11.2865	[6.5235, 11.8915]
$k_{13}$	92.9616	86.3749	22.6051	[16.9820, 91.2826]
$k_{14}$	69.6700	74.2373	0.2118	[0.0664, 67.9548]
$k_{15}$	58.2800	51.5885	48.5051	[45.9896, 70.1724]
$k_{16}$	81.5400	89.7101	76.2469	[68.4823, 95.0394]
$k_{17}$	7.9000	85.8912	0.2689	[0.0213, 7.9164]
$k_{18}$	8.8900	101.0203	14.0437	[9.2273, 16.0937]
$k_{19}$	13.0500	2.9113	8.5301	[0.3245, 12.0491]
$k_{20}$	6.5400	93.7566	9.0591	[4.4476, 12.4538]
$k_{21}$	6.2615	52.6466	0.2456	[0.0223, 7.9409]
$k_{22}$	9.5536	106.5830	15.6348	[5.1120, 16.2136]
$k_{23}$	2.7479	29.7198	0.7268	[0.0109, 5.7346]
$k_{24}$	7.9500	53.8979	13.1923	[8.4006, 19.5529]
$k_{25}$	8.1300	67.2479	4.1898	[1.9219, 12.9717]
$k_{26}$	2.7800	0.0056	0.1197	[0.2465, 4.5403]
$k_{27}$	4.8100	24.7439	9.0857	[5.8291, 13.3738]
$k_{28}$	0.0900	100.8293	3.4949	[0.0506, 1.8452]
$k_{29}$	5.4700	18.1014	0.3826	[0.1967, 1.7861]
$k_{30}$	5.5200	1.7397	0.5090	[0.0.4621, 1.7866]
$k_{31}$	7.8669	4.1334	0.8026	[0.7527, 3.0838]
$k_{32}$	8.6056	5.0141	0.8054	[0.7705, 3.3345]
$k_{33}$	2.6729	1.1504	0.2569	[0.2016, 0.9534]
$k_{34}$	6.6400	56.1957	0.6883	[0.6682, 6.4683]
$k_{35}$	8.3500	21.4817	10.9232	[8.8105, 12.3105]
$k_{36}$	2.6000	60.6397	3.9687	[2.0922, 7.0713]
$k_{37}$	6.0900	68.2011	10.5792	[7.4107, 13.1953]
$k_{38}$	5.9800	71.0124	5.3738	[3.3981, 30.2118]
$k_{39}$	7.0800	90.1295	9.6122	[7.1826, 11.6251]
$k_{40}$	4.2300	97.8241	5.0378	[3.7236, 6.6922]
$k_{41}$	6.9012	77.2770	9.0691	[7.0122, 10.3685]
$k_{42}$	5.1458	55.6608	4.9221	[3.0766, 9.6034]
$k_{43}$	9.8363	94.6713	12.5293	[9.9403, 20.2133]
$k_{44}$	8.0100	55.9309	11.7194	[8.6101, 12.8384]
$k_{45}$	1.7000	5.8116	5.0751	[1.6858, 6.1590]
$k_{46}$	2.0900	10.3061	0.2928	[0.1207, 19.6999]
$k_{47}$	6.2700	87.7272	7.2096	[6.2626, 33.9641]
$k_{48}$	8.4300	48.0844	8.3244	[8.5120, 11.2713]
$k_{49}$	4.4900	84.9190	6.1626	[4.3403, 9.6653]
$k_{50}$	0.9400	1.7720	0.2076	[0.0253, 0.8675]
$k_{51}$	5.2317	51.0507	0.8873	[0.8291, 1.6575]
$k_{52}$	2.8599	62.8507	5.3629	[0.7383, 6.4126]
$k_{53}$	3.2270	2.2573	10.4618	[3.2739, 15.3034]
$k_{54}$	6.4700	1.6055	0.1016	[0.0154, 0.7756]
$k_{55}$	3.2400	1.3503	0.6410	[0.2601, 1.5042]
$k_{56}$	4.9500	2.4355	0.8050	[0.7399, 2.1214]
$k_{57}$	8.9600	5.6437	0.8007	[0.0290, 3.5065]
$k_{58}$	1.2500	0.4722	0.3622	[0.0869, 0.4206]
$k_{59}$	2.3100	0.9681	0.8058	[0.7560, 1.1369]

**Table 7:** The best-fitting parameter values (for both WLS and UWLS approaches) of the Kron reduced mathematical model of the Trypanosoma brucei trypanothione synthetase corresponding to the available time-series experimental data.

Parameters	Estimated Values (UWLS)	Estimated Values (WLS)
$p_{1}$	8.8681	0.3572
$p_{2}$	2.3387	0.5386
$p_{3}$	0.0409	0.0893
$p_{4}$	5.4580	0.9955
$p_{5}$	4.6200	3.0531
$p_{6}$	7.8525	4.9069
$p_{7}$	8.5119	1.9480
$p_{8}$	2.9776	0.6329
$p_{9}$	2.5213	7.1061
$p_{10}$	3.1172	2.7720
$p_{11}$	3.2643	6.1818
$p_{12}$	4.9430	3.6137
$p_{13}$	9.0230	3.2929
$p_{14}$	0.8362	0.5099
$p_{15}$	2.2947	1.5590

**Table 8:** Leave-one-out cross-validation training errors for mathematical model of the Trypanosoma brucei trypanothione synthetase corresponding to unweighted and weighted least squares optimization methods.

	Unweighted Least Squares	Weighted Least Squares
Training error	0.8233	0.6977

## Data Availability

Not applicable.

## Supplementary Materials

The following supporting information can be downloaded at: https://www.mdpi.com/article/10.3390/bioengineering10091056/s1, The MATLAB library corresponding to the parameter estimation method described in the main manuscript.

## Abbreviations

The following abbreviations are used in this manuscript:

CRN	Chemical Reaction Network
CRNT	Chemical Reaction Network Theory
IVP	Initial Value Problem
LOOCV	Leave-One-Out Cross-Validation
MAKRL	Mass Action Kinetics Rate Law
NAR	Nicotinic Acetylcholine Receptors
ODE	Ordinary Differential Equation
TBTS	Trypanosoma Brucei Trypanothione Synthetase
UWLS	Unweighted Least Squares
WLS	Weighted Least Squares
