# Supplementary figures and images for: Parameter Estimation for Kinetic Models of Chemical Reaction Networks from Partial Experimental Data of Species’ Concentrations
Source: Bioengineering (Basel). 2023 Sep 7;10(9):1056. doi: 10.3390/bioengineering10091056 (PMC10526083; doi:10.3390/bioengineering10091056)

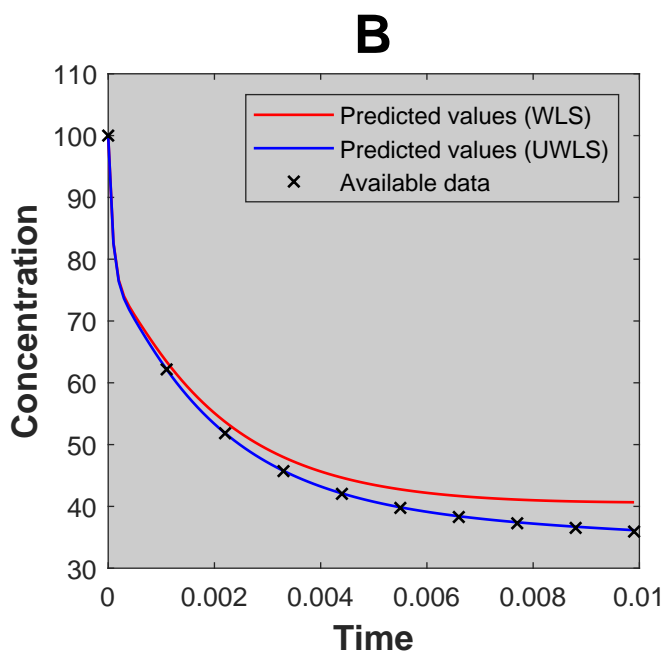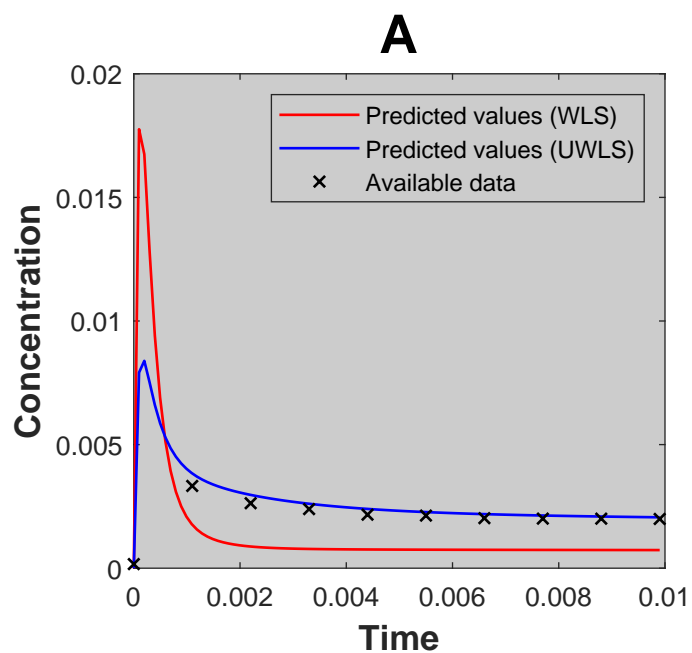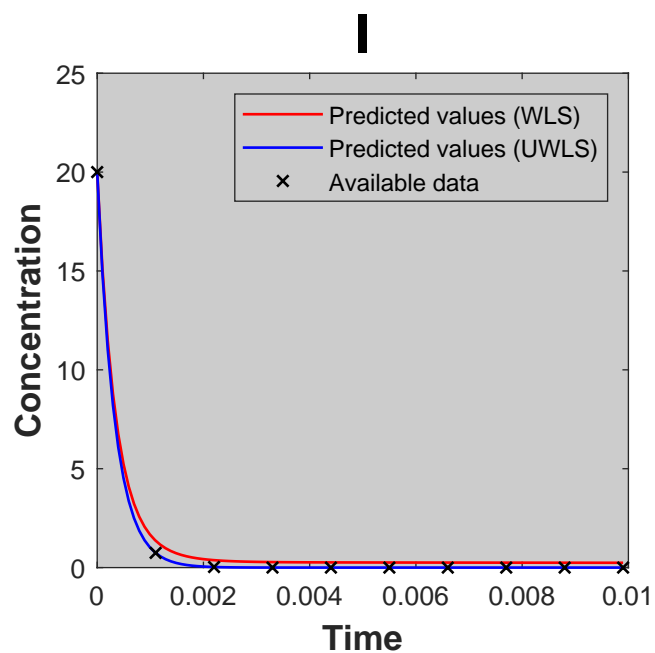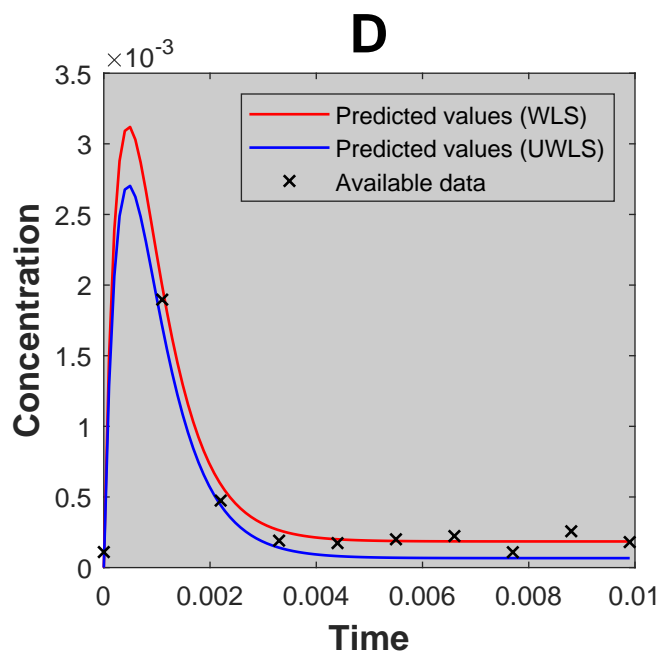

Supplement: Supplementary file 1 [file bioengineering-10-01056-s001.zip › NAR/NARcomparison.pdf]

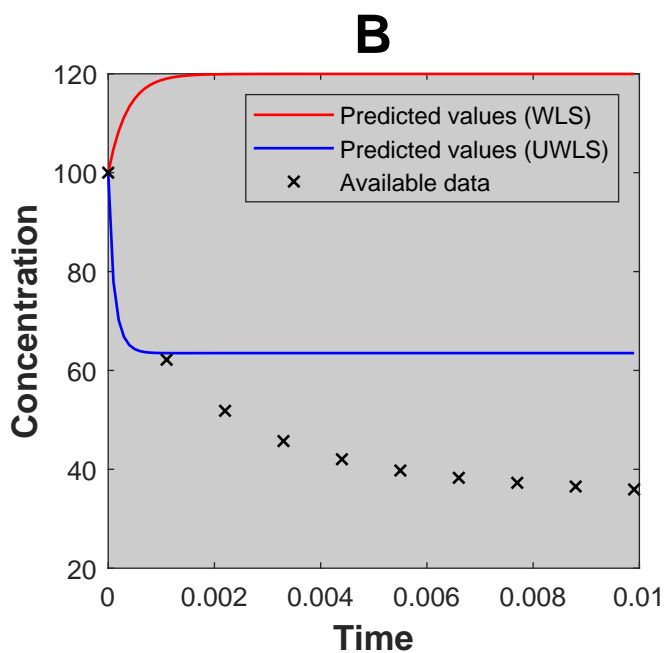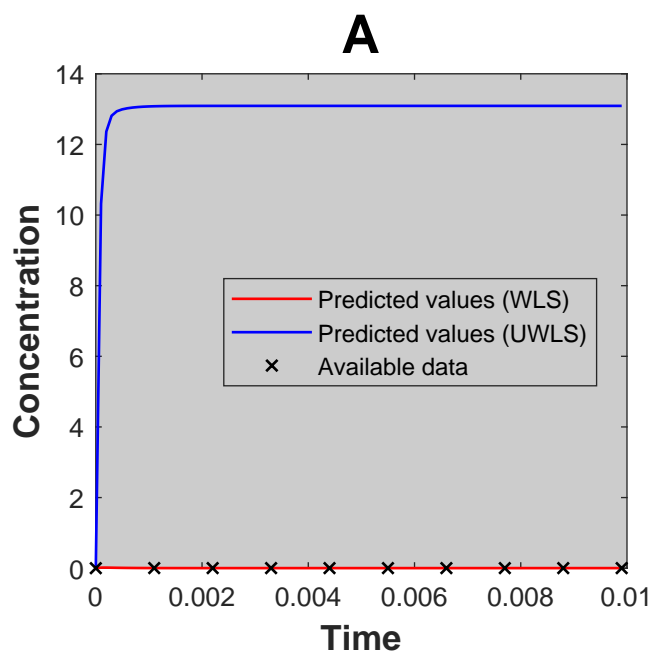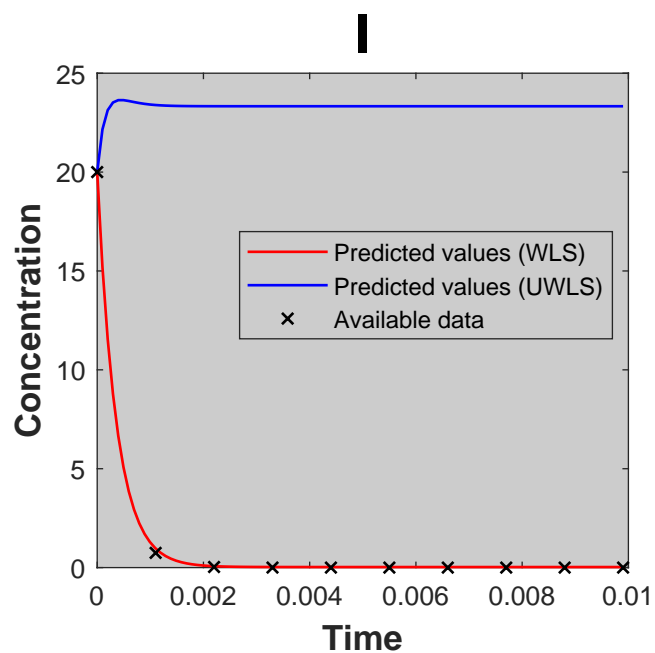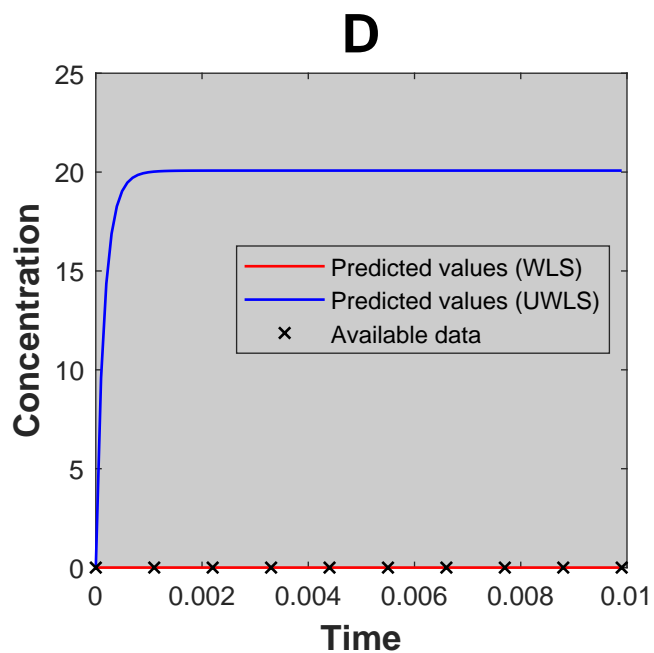

Supplement: Supplementary file 1 [file bioengineering-10-01056-s001.zip › NAR/NARreduced.pdf]
